# Data-informed reconstruction of a bipennate muscle’s aponeurosis and its fibre distribution for performing continuum-mechanical simulations

**DOI:** 10.1007/s10237-025-01989-w

**Published:** 2025-07-22

**Authors:** A. Ranjan, O. Avci, O. Röhrle

**Affiliations:** 1 Fraunhofer Institute for Manufacturing Engineering and Automation IPA, Nobelstr. 12, 70569 Stuttgart, Germany; 2 Institute of Modelling and Simulation for Biomechanical Systems and Cluster of Excellence for Simulation Technology, University of Stuttgart, Pfaffenwaldring 7, 70569 Stuttgart, Germany

**Keywords:** Rectus femoris, Aponeurosis, Diffusion tensor imaging, Finite element method, In-silico analysis, Fibre tractography

## Abstract

Alternatives to Diffusion-Tensor-Imaging tractography methods for determining fibre orientation fields in skeletal muscle include Laplacian flow simulations. Such methods require flux boundary conditions (BCs) at the tendons and/or along the inner aponeuroses, which can significantly influence the gradients of the resulting Laplacian flow. Herein, we propose a novel method based on solving the 3D steady-state thermal heat equations to determine the fibre architecture in a bi-pennate muscle, specifically the *m. rectus femoris*. Additionally, we propose a semi-automated algorithm that provides the geometrical representation of the anterior aponeurosis, which, along with the thermal-based fibre field, is particularly well suited for Finite Element (FE) simulations. The semi-automated reconstruction of the aponeurosis shows a good correlation with manual segmentation, yielding a dice coefficient (DSC) of 0.83. The metamodel-based approach resulted in fluxes with a mean angular deviation of $14.25^\circ \,\pm \,10.36^\circ$ and a fibre inclination from the muscle’s longitudinal axis of $0.44^\circ \,\pm \,4.48^\circ$. Comparing the mechanical output of the same *m. rectus femoris* muscle geometry informed by the two respective fibre architectures showed that the most significant contributing factor was the relative fibre inclination. Compared to the standard deviation in the undeformed configuration ($0.44^\circ \,\pm \,4.48^\circ$), the standard deviation of relative fibre inclination during passive stretching at low applied loads, for instance, at $30\%$ of the maximum applied load, showed a significant decrease ($0.49^\circ \,\pm \,2.24^\circ$). Similarly, at maximum isometric contraction, the relative fibre inclinations at $10\%$ initial fibre pre-stretch are $0.19^\circ \,\pm \,1.23^\circ$, indicating a drop in standard deviation from the undeformed configuration ($0.44^\circ \,\pm \,4.48^\circ$). The current study demonstrates that despite the initial deviations in fibre orientations and relative fibre inclinations, thermal flux-based fibre orientations not only exhibit comparable results to DTI-based fibre tractography for the macroscopic analysis of the *m. rectus femoris* but also result in homogeneous stretch fields and improved numerical convergence. The proposed methods may be applied to determine inner aponeuroses of other bi- or multi-pennate muscles, enabling efficient in-silico computations of the musculoskeletal system.

## Introduction

To fully leverage the potential of in-silico models for analysing the biomechanics of movement at the individual level, there is a critical need to personalise our skeletal muscle models and enhance our comprehension of muscular physiology. State-of-the-art simulations in movement biomechanics typically model the skeletal muscles’ mechanical behaviour using lumped-parameter models such as the classical three-element Hill-type muscle models (Hill 1938; Zajac 1989; Winters 1990). In these models, the mechanical behaviour of the respective muscles is described using a contractile element (CE), a series elastic element (SEE), and a parallel elastic element (PEE). Within these elements, the complex and heterogeneous overall mechanical behaviour is described using lumped parameters valid for all muscles. These parameters might be informed by basic anatomical characteristics, such as the (physiological) cross-sectional area or the (over the entire muscle averaged) pennation angle (Kellis et al. 2012; Lee et al. 2015; Sopher et al. 2017), and hence, not on an individual basis. For applications in which the muscular anatomy plays a crucial role, the lumped-parameter models are only of limited applicability. On the other hand, continuum mechanical models can take into account spatial dimensions and material heterogeneities. Using the governing equations of nonlinear elasticity, which are discretised using the finite element method (FEM), these models have emerged over the last two decades and can integrate various spatially heterogeneous skeletal muscle properties, e.g., realistic and complex muscle fibre architecture (Blemker and Delp 2005; Handsfield et al. 2017), motor-unit-guided activation dynamics (Heidlauf and Röhrle 2014; Giantesio and Musesti 2017), vivid anatomical features (Röhrle et al. 2017; Mo et al. 2018; Ramasamy et al. 2018; Avci and Röhrle 2024) under complex loading scenarios (Röhrle and Pullan 2007; Stavness et al. 2012; Zeng et al. 2023) in a straightforward manner. The detail and complexity of the musculoskeletal systems modelled in these studies exhibit significant variability. For example, the study by Handsfield et al. (2017) focuses exclusively on determining the fibre architecture of the *m. gastrocnemius* muscle, whereas the limb-socket interaction study by Ramasamy et al. (2018) addresses only the non-contractile behaviour of skeletal muscles. Conversely, the lower limb investigation by Mo et al. (2018) captures the contractile behaviour of skeletal muscles, while the study by Avci and Röhrle (2024) on the upper extremity incorporates both nonlinear passive and active behaviours of skeletal muscles, including the influence of initial stretch levels on deformation of skeletal muscles. Nevertheless, given that skeletal muscle tissue is a highly anisotropic material, the predictive accuracy of continuum-mechanical skeletal muscle models is contingent upon their ability to realistically represent internal anatomical features such as the muscle fibre architecture. For bi- or multi-pennate skeletal muscles, this also includes the mechanical and geometric representation of the aponeurosis, which has been neglected in almost all existing (continuum-mechanical) skeletal muscle models, e.g., (Röhrle and Pullan 2007; Röhrle et al. 2017; Avci and Röhrle 2024).

As far as information on muscle fibre architecture is concerned, it has typically been obtained from cadaver dissections (Friederich and Brand 1990; Wickiewicz et al. 1983). More recent methods to obtain more detailed information on the actual muscle fibre architecture include, but are not limited to, X-ray-based in vitro methods for skeletal muscle (Kupczik et al. 2015) or contrast tomography-based methods for high-resolution imaging of cardiac muscle (Brunet et al. 2024), in-vivo magnetic resonance (MR) spectroscopic imaging (Vermathen et al. 2003) including diffusion tensor imaging (DTI) (Damon et al. 2011; Froeling et al. 2012), or ultrasound imaging (Rana et al. 2009; Cunningham et al. 2017; Zheng et al. 2021; Sahrmann et al. 2024). Although some data originating from such imaging methodologies, particularly DTI data, might be suitable for fibre tractography- a promising and successfully used technique in neuroimaging (Jelescu and Budde 2017)- one cannot directly transfer the (general) parameters for fibre reconstruction used in neurology to musculoskeletal studies. The structure and physiology of these two tissues are inherently too different. Extracting anatomical features from skeletal muscles using DTI exhibits several disadvantages, e.g., in terms of acquisition time, low signal-to-noise ratio (SNR), etc. and the acquired sequences additional de-noising and corrections for motion or eddy currents (Andersson and Sotiropoulos 2016). This is time-consuming and, hence, expensive, limiting their application for determining anatomical features of skeletal muscle tissue. A further limiting factor of using fibre information directly extracted from the DTI data and imported into continuum-mechanical simulations is the fact that fibre orientations obtained by fibre tractography data can locally exhibit strong irregularities (in particular at the muscle boundaries), leading to non-continuous fibre fields and subsequently to numerical issues such as instability, non-convergence, or stress/strain localizations. To avoid these, purely numerical approaches like template mapping or the computation of Laplacian vector fields have been introduced. For a more detailed review of using DTI to extract muscle fibre information, see Heemskerk and Damon (2007) and Oudeman et al. (2016).

The method of approximating fibre orientations using Laplacian vector fields has been widely applied in cardiac muscle studies. In the research by Wong and Kuhl (2014), the Poisson equation was employed with isotropic diffusion coefficients and Dirichlet boundary conditions (BCs) to generate fibre orientations for a human heart model. The generated orientations were compared with analytically derived ones, resulting in an error of less than $20\%$ for most fibres. Bayer et al. (2012) introduced a novel Laplace–Dirichlet Rule-Based (LDRB) algorithm for generating myocardial fiber orientations and conducted simulations of electrical activation using both DTI-based and LDRB-based orientations. Their comparative analysis demonstrated strong agreement between LDRB-based and DTI-based fibres, with mean angular differences of $23^{\circ }$ and $38^{\circ }$ in the transverse and longitudinal directions, respectively, and a mean difference of $3.15\,\textrm{ms}$ in activation times. For a comprehensive review of LDRB methods and their application in determining myocardial fiber orientations, see Piersanti et al. (2021). Beyond the myocardium, the Laplacian approach has also been utilised in other fibrous tissues, such as the human tongue. Gomez et al. (2018) found that Laplacian-based fibres exhibited a mean angular difference of $22^{\circ }$ compared to DTI-based fibres and revealed strain differences ranging from $0.4\%$ to $20\%$ in structural analyses between DTI-based and Laplacian-based fibre directions.

As far as template mapping methodologies for modelling fibre orientations in skeletal muscles are concerned, they are based on macroscopic information, e.g., pennation type and overall anatomical shape of the muscles, and transformations of pre-existing sets of artificial fibre templates (Blemker and Delp 2005; Kohout and KukaÄka 2014). As this methodology considers the anatomical shape of a muscle and some lumped information, it already provides some degree of subject-specificity. Although such methods are numerically effective, they are limited by the overall number of unique fibre templates. On the upside, they produce approximative fibre fields without DTI data. In most cases, they fall short in modelling the internal structures like the aponeuroses as 3D geometrical entities. A few notable exceptions are the studies by Rehorn and Blemker (2010) on the *m. biceps femoris* muscle and Diniz et al. (2023, 2024) on the *m. gastrocnemius* muscle.

Previous studies that have employed Laplacian vector field approaches for skeletal muscles have similar shortcomings. In the study by Choi and Blemker (2013), a streamline tracing method for divergence flow fields was implemented, a method first introduced for skeletal muscle tissues by Klausen et al. (2012). Inouye et al. (2015) expanded this work by introducing flow guides, which were generated by an incompressible, viscous, laminar computational fluid dynamics (CFD) simulation. Handsfield et al. (2017) were the first to validate the outcome of such CFD simulations against DTI-based fibre trajectories by highlighting their differences. Here, the authors reported high sensitivity of simulation results to the aponeurosis location and modest sensitivity to the mesh density. Varvik et al. (2021) showed the angular deviations of two fields, namely, Laplacian and Stokes fields, from DTI-based fibre tractography and performed structural FE simulations, once based on DTI-derived and once from a CFD-derived fibre orientations. The results of the FE analysis exhibited relatively similar strain distributions with minor variations in stress concentrations at certain localised regions in the proximal and laterodistal regions of the muscle. Despite their relative success at approximating the fibre architecture from DTI datasets, all these methodologies share the same limiting factors as the template mapping approaches, as they lack the geometrical representation of internal structures such as the aponeurosis.

In more recent studies involving the *m. gastrocnemius* muscle (Diniz et al. 2023, 2024), the isometric contraction of the muscle was simulated by applying external forces to the cross section of the muscle and the outer surface of the tendon. The aponeurosis of the *m. gastrocnemius* muscle was modelled as an internal FE structure using MRI sequences, and the fibre orientations were generated using the methodology proposed by Choi and Blemker (2013). Nevertheless, this study also had similar limiting factors as the template mapping approaches, including the additional assumption of abstracting the constitutive modelling of active skeletal muscles through external forces.

The current study draws on the findings of Handsfield et al. (2017). There, the resulting fibre orientations were shown to depend on the positioning and geometry of the aponeurosis. Herein, we elevate this shortcoming by presenting a novel procedure to model the location and structure of the aponeurosis within bi-pennate muscles and use, instead of CFD simulations, the 3D heat equations of thermal conduction to obtain a numerical representation of the fibre field. Instead of (manually) segmenting the aponeurosis from high-resolution MRI or ultrasound imaging sequences (Barnouin et al. 2014), we apply vector operations on the fibre orientation field estimated from the DTI data to isolate voxels that are at the centre of the aponeurosis. We choose the *m. rectus femoris* to exemplify our new methodology by reconstructing and modelling its aponeurosis. Our 3D heat equation approach offers considerable computational advantages over fluid simulations, e.g., steady-state thermal simulations depend only on thermal gradients controlled by BCs while the streamlines obtained by Stokes flow directly depend on the applied BCs (Varvik et al. 2021). For the thermal simulation-based approach, we could determine optimal BCs that resulted in a fibre orientation field close to the DTI one by using a metamodel-based optimisation, minimising the angular deviations between heat flux vectors and DTI-based fibre orientations. The fibre orientations derived from the optimisation and DTI scans are used in continuum-mechanical simulations of skeletal muscle tissue subject to different loading conditions, and its mechanical output is compared and analysed.

In summary, (i) we introduce an imaging-based algorithm to identify the geometry of the aponeurosis and the muscle fibre orientation. We demonstrate its feasibility (ii) using a geometrical FEM model of the *m. rectus femoris* by performing a sensitivity study that analyses the influence of varying thermal gradients in the muscle and aponeurosis boundaries on estimating optimal muscle fibre orientations. Further, (iii) compare the mechanical behaviour of the muscle–tendon–aponeurosis (MTA) complex modelled with (a) DTI-based fibre orientations and (b) optimised heat flux fibre vectors. For both cases, we use the same volumetric model of a *m. rectus femoris* and the same transversely isotropic constitutive model, passive stretch, and isometric contraction condition. As a measure of accuracy, we compare the differences between the fibre stretches obtained from structural FE simulations. The outcome of this research is a continuum-mechanical model of the bi-pennate *m. rectus femoris* muscle with a smooth and realistic muscle fibre field and a realistic and volumetric description of its anterior aponeurosis. This enables robust simulations and detailed continuum-mechanical analysis of the structural behaviour of the *m. rectus femoris*. The proposed methodology is general in nature and applies in the same way to bi- and multi-pennate muscles other than the *m. rectus femoris*.

## Methods and models

Identifying and segmenting an aponeurosis from imaging data is often challenging and cumbersome. Even segmenting the aponeurosis from high-resolution MRI data is highly subjective due to its thin structure and the potential presence of connective and fat tissue surrounding it. We propose a novel fibre-tractography-based method to identify and reconstruct its geometrical representation for muscles with aponeurosis. Further, a realistically reconstructed fibre orientation shall be the basis for robust continuum-mechanical simulations of the muscle’s structural behaviour. Although the proposed methodology also applies to muscles with more than one aponeurosis, we demonstrate the methodology and feasibility on the *m. rectus femoris* and its anterior aponeurosis.

Based on MRI and DTI data of the same *m. rectus femoris* (cf. Sect. 2.1), we introduce a novel methodology to automatically determine the geometry of the muscle’s anterior aponeurosis including the geometrical representation of the MTA-complex (cf. Sect. 2.2). The results are CAD- and FEM-models of our targeted muscle. Section 2.3 introduces a novel concept of using the heat equation to automatically generate a consistent and realistic muscle fibre field. Section 2.4 describes the constitutive laws for the different MTA-complex structures and defines several load case scenarios for structural analysis. These are used to demonstrate the advantages of our novel method over DTI-based fibre architecture.

### Acquisition of imaging datasets

**Fig. 1:**
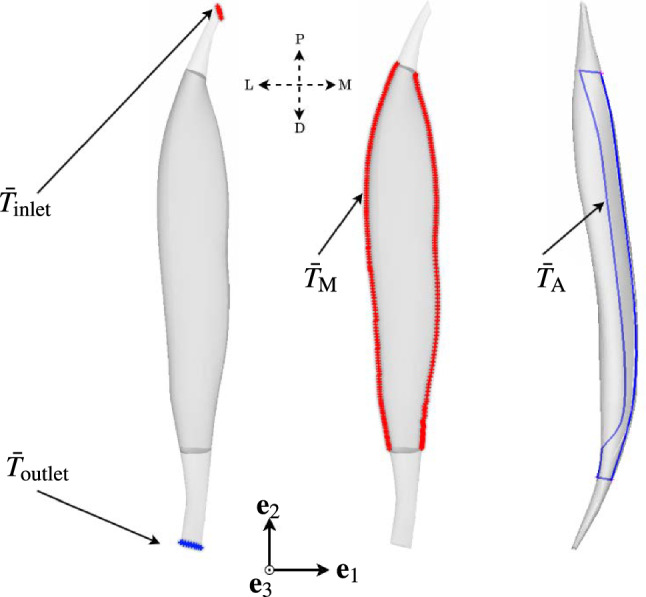
Boundary conditions of the thermal simulation with fixed temperature values $\bar{T}_\mathrm {{inlet}}$, $\bar{T}_\mathrm {{outlet}}$ on proximal and dorsal ends respectively (left) and temperature fields on sub-domains, $\bar{T}_\textrm{M}$ (middle) and $\bar{T}_\textrm{A}$ (right). The letters P, D, L and M refer to anatomical directions proximal, distal, lateral and medial, respectively

Imaging datasets were acquired out on a 42 year old male subject at University Hospital Tübingen, Germany using a 3T MRI (Magneton Prisma, Siemens Healthineers) scanner (ethics approval ID S-850/2019). T1-sequences were acquired using the following parameters: echo time (TE) $11\,\textrm{ms}$, repetition time (TR) 700 ms, flip angle (FA) 120$^{\circ }$, in-plane resolution 1.1 $\times$ 1.1 mm$^{2}$, acquisition matrix 384 $\times$ 384, slice thickness 1.1 mm with 448 coronal slices. Additionally, the parameters of the DTI sequences were: TE 30 ms, TR 10,000 ms, Bandwidth 2405 Hz/Px, in-plane resolution 2.625 $\times$ 2.625 mm$^{2}$, slice thickness 6.25 mm with 12 diffusion directions with b-values ranging between 0 and 700 s/mm$^{2}$. Eddy current distortions and additional noises were removed using the model free prediction methodology (Andersson and Sotiropoulos 2016) and local principal component analysis based de-noising algorithm (Manjón et al. 2013), respectively. For modelling purposes, we define a new global coordinate system, in which the $\textbf{e}_2$ component is aligned with principal muscle orientation, while $\textbf{e}_1$ and $\textbf{e}_3$ are orthonormal to each other, spanning a plane orthogonal to $\textbf{e}_2$ (cf. Fig. 1). The image coordinate systems of the MRI and DTI data set can then easily be transformed into the newly defined global coordinate system through rigid-body transformations.

### Automatic generation of internal muscle architecture

#### Fibre density based estimation of aponeurosis

Based on the primary diffusion direction of water molecules along neural tracts or muscle fibres/fascicles, fibre tractography can estimate the underlying 3D fibre orientation field (Damon et al. 2011). The fibre orientation field is computed using either a deterministic or probabilistic algorithm on a set of pre-defined seed points (Garyfallidis et al. 2014). The most important tracking parameter is the fractional anisotropy (FA), which describes the preferred direction of water diffusion in skeletal muscles and is given by:

1 $$\begin{aligned} \textrm{FA} = \frac{1}{2}\frac{\sqrt{(\Lambda _1-\Lambda _2)^2 + (\Lambda _2-\Lambda _3)^2 + (\Lambda _3-\Lambda _1)^2}}{\sqrt{\Lambda _1^2+\Lambda _2^2+\Lambda _3^2}}, \end{aligned}$$

where $\Lambda _1$, $\Lambda _2$ and $\Lambda _3$ are eigenvalues of the diffusion tensor.

The properties of further tracking parameters are based on the definitions given by Bolsterlee et al. (2018, 2019) and restrict the FA to ranges between 0.1 and 0.5 ($0.1\le \text{ FA }\le 0.5$), limits the fibre turning angle to a maximum of $10^{\circ }$ and only considers fibre tracts that are between $15\,\textrm{mm}$ and $200\,\textrm{mm}$ in length.

To define the averaged fibre orientation of a voxel *V*, we define a sphere around the centre point of each voxel *V* with diameter $2.625\,\textrm{mm}$ and count the number of fibres passing through this sphere. The diameter of the sphere is chosen such that it is completely enclosed in the (not cubic) voxel, which has a volume of 2.625 $\times$ 2.625 $\times$ 6.25 mm$^{3}$. We define *K*(*V*) as the number of fibres passing through the sphere centred in voxel *V*. Then, for each voxel *V*, its averaged and representative orientation vector $\hat{\textbf{a}}_0^V$ is computed by

2 $$\begin{aligned} \hat{\textbf{a}}_0^V = h(V)\frac{1}{K(V)}\sum _{k=1}^{K(V)} \textbf{a}_{0,k}^V, \end{aligned}$$

where $\textbf{a}_{0,k}^V$ denotes all the fibres within the embedded sphere and *h*(*V*) is defined as

3 $$\begin{aligned} \begin{aligned}&h(V)= {\left\{ \begin{array}{ll} 1,& \big [\sum _{k=1}^{K}\textbf{a}_{0,k}\big ]\cdot \textbf{e}_2\ge 0,\\ -1,& \text {otherwise}, \end{array}\right. }\\ \end{aligned} \end{aligned}$$

and defines the overall orientation of the averaged orientation vector.

In muscles with aponeurosis, fibres span from the tendon to the aponeurosis. Hence, an aponeurosis acts as a potential source or sink term in such muscles. This implies that the *divergence* of the vector field $\hat{\textbf{a}}_0^V$ for the region spanning the entire MTA-complex should be zero. The divergence can be approximated by its discrete version in which we sum up the normalised vector orientations over a region of interest, i.e. summing up the number of fibres entering (+ 1) and leaving (− 1). This discrete *divergence* provides a measure to identify fibre sources and sinks within the muscle tissue. Furthermore, counting the number of unique fibres *K* within voxel *V* results in a density map of the muscle.

Within our proposed new methodology, we define a region of interest (RoI) which excludes the posterior voxels of the muscle and hypothesize that the anterior aponeurosis is located in areas where high local *divergence* and high-density map values coincide. For our work, we identified voxels as a potential aponeurosis location if its values exceed a threshold of 3.0 for the *discrete divergence* value and 600 for the density map value. Outlier voxels were filtered out by first determining all connected regions (Wu et al. 2005) and then filtering out islands that contain less than 10 voxels. Consequently, the largest connected component in the core of the tissue yields the desired geometrical representation of the aponeurosis.

#### MRI-based generation of skeletal muscle geometry

The volumetric representation of the bi-pennate muscle and associated tendons is obtained via manual segmentation of MRI sequences. We utilised Simpleware ScanIP R-2021.03 (Synopsys, Sunnyvale, US), employing generic threshold segmentation techniques to highlight boundaries within muscle groups. Tendons are segmented manually, whereas the anterior aponeurosis is identified as described in Sect. 2.2.1. The segmented volumes are then meshed using the internal meshing algorithm of Simpleware, with the following mesh parameters: coarseness $-10$, number of optimization cycles 5 and mean and minimum Jacobian values of 0.6 and 0.2, respectively. This procedure generates a volumetric mesh comprising $121\,516$ linear tetrahedral elements. However, global meshing operations may introduce discontinuities and low-quality elements, particularly at the muscle-tendon and muscle-aponeurosis interfaces. We reconstructed the volumetric mesh using $\textrm{C}^2$-continuous CAD entities, i.e., B-splines and NURBS to ensure geometric continuity across these interfaces. These entities are manually generated using ANSA v20.1.0 (Cadence Design Systems, San Jose, US). After the CAD-based reconstruction, the encompassing NURBS surfaces have been re-meshed with triangular elements adhering to the following target meshing parameters: minimum and maximum element lengths are $1.5\,\textrm{mm}$ and $3.0\,\textrm{mm}$, respectively, a the Jacobian of the elements must be larger than 0.7, and minimum and maximum triangular angles are $35^{\circ }$ and $70^{\circ }$, respectively. The final volumetric mesh is derived from the surface mesh using a growth rate of 1.2 and a maximum allowable aspect ratio of 2.0. An illustration of the CAD- and FE-model of the MTA-complex along with the fibre orientations is provided in Fig. 6.

### Automatic generation of muscle fibre orientation field

#### 3D heat equations for thermal conduction

Again, we exemplify the generation of a realistic muscle fibre orientation field for the *m. rectus femoris*. This method, however, is general and could be applied to any other (bi- or multi-pennate) muscle.

The governing equations of heat conduction at a given point $\textbf{x}$, time *t* and temperature field $T(\textbf{x},t)$ is governed by:

4 $$\begin{aligned} \left. \begin{aligned}&\rho c \frac{\partial T(\textbf{x},t)}{\partial t} = -\nabla \cdot (\textbf{q}_h(\textbf{x},t)), \\ \end{aligned} \right. \end{aligned}$$

where $\rho$ and *c* represents the mass density and heat capacity respectively, while the symbol ($\partial$) and ($\nabla \cdot$) represent the partial derivate and the divergence of a vector field, and heat flux, $\textbf{q}_h(\textbf{x},t)$. The constitutive equation governing the heat flux is a function of the temperature gradient, i.e.,

5 $$\begin{aligned} \left. \begin{aligned}&\textbf{q}_h(\textbf{x},t) = -\kappa \nabla T(\textbf{x},t), \end{aligned} \right. \end{aligned}$$

where $\kappa$ is the thermal conductivity. Under steady state conditions, the transient parameters drop out and Eq. (4) reduces to:

6 $$\begin{aligned} \left. \begin{aligned}&\nabla \cdot (\nabla T(\textbf{x})) = 0.\\ \end{aligned} \right. \end{aligned}$$

Herein, the unit directions of heat fluxes derived from a steady state, 3D-thermal simulation are used as an alternative to DTI-based fibre tractography. It is assumed that variations in thermal tractography arise only due to spatial changes within the thermal boundary conditions. Figure 1 depicts the applied boundary conditions. The figure on the left depicts the temperature boundary conditions at the respective distal and proximal ends of the bone-tendon interface, i.e., $\bar{T}_\textrm{inlet}$ and $\bar{T}_\textrm{outlet}$, the centre one visualises in red the muscle boundary, $\bar{T}_\textrm{M}$, and the right one highlights in blue the tendon-aponeurosis boundary, $\bar{T}_\textrm{A}$. Temperature boundary conditions are applied to lateral and medial boundaries of the muscle and selected faces across the central axis of the aponeurosis extending up to the distal end of the muscle, along with terminal faces of the proximal and dorsal tendons. Constant temperature values are defined for both terminal faces of the tendons. Further, appropriate temperature gradients must be identified to generate a bi-pennate-like orientation of heat flux vectors. As such, we apply different temperature gradients to boundaries $\bar{T}_\textrm{M}$ and $\bar{T}_\textrm{A}$. This ultimately leads to heat fluxes directed towards the aponeurosis of the muscle. We summarise the applied boundary conditions at the bone-tendon ends as follows. Let

7 $$\begin{aligned} \left. \begin{aligned} \bar{T}_\textrm{inlet} = T_\textrm{0}\quad \textrm{and} \quad \bar{T}_\textrm{outlet} = T_\textrm{1},\\ \end{aligned} \right. \end{aligned}$$

with $T_0$ and $T_1$ being constant temperature values. The boundary conditions for the muscle and aponeurosis boundary surfaces are defined as:

8 $$\begin{aligned} \left. \begin{aligned} \bar{T}_\textrm{M}\;:= \bar{T}_\textrm{M}(n_z^\textrm{M})\quad \textrm{and} \quad \bar{T}_\textrm{A}\;:= \bar{T}_\textrm{A}(n_z^\textrm{A}), \end{aligned} \right. \end{aligned}$$

respectively, where $n^\textrm{M}_z$ and $n^\textrm{A}_z$ are normalised values spanning the respective boundary surface. They are defined by

9 $$\begin{aligned} \left. \begin{aligned}&n_z^\textrm{M} = \frac{z^\textrm{M} - z_{\textrm{min}}^\textrm{M}}{z_{\textrm{max}}^\textrm{M} - z_{\textrm{min}}^\textrm{M}}\quad \text {and}\\&n_z^\textrm{A} = \frac{z^\textrm{A} - z_{\textrm{min}}^\textrm{A}}{z_{\textrm{max}}^\textrm{A} - z_{\textrm{min}}^\textrm{A}}, \end{aligned} \right. \end{aligned}$$

respectively. In Eq. (9), $z_{\textrm{max}}^\textrm{M}$, $z_{\textrm{max}}^\textrm{A}$ are the maximum global coordinates, whereas, $z_{\textrm{min}}^\textrm{M}$, $z_{\textrm{min}}^\textrm{A}$ are the minimum global coordinates of $z^\textrm{M}$ and $z^\textrm{A}$ along axis $\textbf{e}_2$ of boundaries, $\bar{T}_\textrm{M}$ and $\bar{T}_\textrm{A}$, respectively. Then, the specific scalar field equations, $\bar{T}_\textrm{M}(n_z^\textrm{M})$, $\bar{T}_\textrm{A}(n_z^\textrm{A})$ are given by the following relationships:

10 $$\begin{aligned} \left. \begin{aligned}&\bar{T}_\textrm{M}(n_z^\textrm{M}) = T_0(n^\textrm{M}_z)^{\alpha _1}\quad \text {and}\\&\bar{T}_\textrm{A}(n_z^\textrm{A}) = T_0(n^\textrm{A}_z)^{\alpha _2}, \end{aligned} \right. \end{aligned}$$

where $\alpha _1$ and $\alpha _2$ are exponential constants. The parameters and constants introduced in Eqs. (7–10) are defined in the next section.

Note, applying only the boundary conditions provided in Eq. (7) by itself would lead to a fusiform vector field. Only the addition of Eqs. (7) and (8) turn the resulting vector field into a feather-like vector orientation – the vector field that we then associate and use for describing bi-pennate skeletal muscle fibre fields.

#### Identification of parameters of thermal boundary conditions

**Table 1:** Initial values and ranges of parameters $T_1, \alpha _1$ and $\alpha _2$ for generation of parametric design space ${\textbf {D}}$

Parameter	$T_1$	$\alpha _1$	$\alpha _2$
Design space	$-200\le -100.0 \le -1$	$0.0\le 5.0 \le 20.0$	$0.0\le 10.0 \le 20.0$

Here, the negative values of temperature $T_1$ do not have a physical significance but are used to generate appropriate thermal gradients within the muscle

**Fig. 2:**
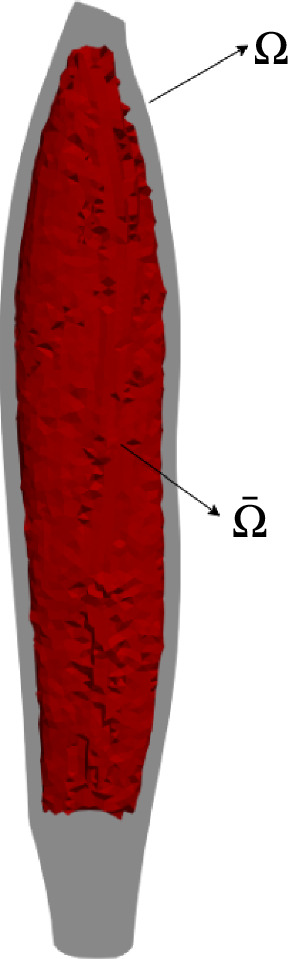
Domain of the MTA-complex $\Omega$ and sub-region $\bar{\Omega }$ defined for the identification of parameters of thermal BCs

To minimise the deviations between the vector field obtained as a solution for the thermal flow simulations and the fibre orientations determined by DTI-based tractography, we formulate an optimisation problem. In the case that the DTI-based fibres reconstruction contains regions with chaotic fibre orientations or regions influenced by surrounding tissue, we restrict our optimisation problem to a subset $\bar{\Omega }$ of the entire MTA-complex domain $\Omega$, excluding those regions. In our case, these were the distal region of the *m. rectus femoris* and the region corresponding to the muscle’s exterior surface defined by a thickness of 3 mm, i.e., the thickness of a single tetrahedral element. As a result, sub-region $\bar{\Omega }$ contains $36\%$ lesser elements than region $\Omega$. Subsequently, the terms introduced in the following section that correspond to the region $\bar{\Omega }$ are characterised by a $\bar{(\cdot )}$. The regions $\Omega$ and $\bar{\Omega }$ are illustrated in Fig. 2.

The optimisation problem itself is described by:

11 $$\begin{aligned} \textbf{s}_{\textrm{opt}}=\text{ arg }\!\min \bar{y}_{\bar{\Omega }}(\textbf{s})\, \quad \forall \textbf{s}=[T_1, \alpha _1, \alpha _2] \in \textbf{D}. \end{aligned}$$

Herein, the 3D design space $\textbf{D}$ is composed of parameters $T_1$, $\alpha _1$ and $\alpha _2$ from Eqs. (7) and (8). The range of parameters is given as follows: (i) minimum and maximum values of $T_1$ are $-200$ and $-1$ respectively, (ii) minimum and maximum values of $\alpha _1$ and $\alpha _2$ are 0.0 and 20.0 respectively. The starting values of $T_1$, $\alpha _1$ and $\alpha _2$ are set to $-100.0$, 5.0 and 10.0, respectively. It is to be noted that the temperature values have no physical significance but are only used to generate thermal gradients within the FE-model. The optimization is constrained in the region $\alpha _2 - \alpha _1 \ge 0$. The constraint ensures that the resulting heat vectors align from the medio-superior or latero-superior directions towards the centre, i.e., towards the location of the anterior aponeurosis of the *m. rectus femoris*. The inlet temperature $T_0$ from Eq. (7) is set at a constant value, i.e. $1\,\textrm{K}$, and is therefore not included in $\textbf{D}$. The objective function minimises the averaged angular deviations between the fibre orientations obtained from the DTI tractography and the solution of the thermal heat equation. For each element *k*, the angular deviation $\bar{\Omega }^k \in \bar{\Omega }$ is defined by the cosine distance

12 $$\begin{aligned} y^k(\textbf{s}) = 1-\frac{\textbf{a}_{\textrm{therm}}^{k}(\textbf{s}) \cdot \textbf{a}_{\textrm{DTI}}^{k}}{\Vert \textbf{a}_{\textrm{therm}}^{k}(\textbf{s})\Vert \Vert \textbf{a}_{\textrm{DTI}}^{k}\Vert }, \end{aligned}$$

where $\textbf{a}_{\textrm{DTI}}^{k}$ and $\textbf{a}_\textrm{therm}^{k}(\textbf{s})$ are the vectors of the respective DTI-based and thermal-simulation-based fibre orientation obtained for design variable $\textbf{s}$ in $\bar{\Omega }^k \in \bar{\Omega }$. The averaged angular deviation overall *k* constituting $\bar{\Omega }$, i.e., $\bigcup _{k=1}^{K} \bar{\Omega }^k = \bar{\Omega }$, is then computed as its geometrical mean, i.e.,

13 $$\begin{aligned} \bar{y}_{\bar{\Omega }}(\textbf{s}) = \frac{1}{K}\sum _{k=1}^{K} y^k(\textbf{s}). \end{aligned}$$

To solve the optimisation problem, we chose for $\textbf{s}\in \textbf{D}$ a total of $N_{\textbf{D}} = 200$ design points, Radial Basis Function (RBF) networks, and the Adaptive Simulated Annealing (ASA) algorithm in LS-OPT (Stander et al. 2020). The design space’s parameters and ranges are tabulated in Table 1.

### Mechanical analysis of MTA-complex

#### Continuum model for skeletal muscles

The MTA-complex is simulated using a three-dimensional, continuum-mechanical approach. The skeletal muscle tissue, tendon, and aponeurosis are modelled using a hyperelastic, transversely isotropic material model with active and passive force components. We choose an additive split of the active and passive stresses to model the active behaviour of the skeletal muscle tissue (Röhrle et al. 2017).

**Table 2:** Material parameters of the soft tissue model for the MTA-complex

Model	Material	Type	Value
Muscle	${C}^{\text{ M }}_{\text{1 }}$	Isotropic	2e-3 MPa
	${C}^{\text{ M }}_{\text{2 }}$	Isotropic	2e-4 MPa
	*k*	Isotropic	7.2 MPa
	${C}^{\text{ M }}_{\text{3 }}$	Fibre (passive)	5e-3 MPa
	${C}^{\text{ M }}_{\text{4 }}$	Fibre (passive)	6 (–)
	$\gamma$	Muscle	0 (–)
	$\sigma _\textsf {max}$	Fibre (active)	0.3 MPa
	$\Delta W_\textsf {asc}$	Fibre (active)	0.15 (–)
	$\Delta W_\textsf {dsc}$	Fibre (active)	0.16 (–)
	$\nu _\textsf {asc}$	Fibre (active)	2 (–)
	$\nu _\textsf {dsc}$	Fibre (active)	6 (–)
	$\Lambda _\textsf {opt}$	Fibre (active)	1.3 (–)
Tendon and aponeurosis	${C}^{\text{ T }}_{\text{1 }}$	Isotropic	2e-2 MPa
	${C}^{\text{ T }}_{\text{2 }}$	Isotropic	2e-3 MPa
	${C}^{\text{ T }}_{\text{3 }}$	Fibre (passive)	0.5 MPa
	${C}^{\text{ T }}_{\text{4 }}$	Fibre (passive)	25 (–)
	$\gamma$	Tendon	1 (–)

Based on the strain-energy formulation by Crisfield (1997) for an isotropic, quasi-compressible Mooney–Rivlin-type material, Avci and Röhrle (2024) described the isotropic second Piola–Kirchhoff stress tensor for representing the soft tissue matrix $\textbf{S}_\textrm{iso}$ as follows:

14 $$\begin{aligned} \textbf{S}_{\textrm{iso}} = (B_1\textbf{I}+B_2\textbf{C}+B_3\textbf{C}^{-1})+k\,(\textrm{det}\,\textbf{F}-1)I_3^{1/2}\textbf{C}^{-1}\,, \end{aligned}$$

where *k* gives the bulk modulus of soft tissue matrix, and,

15 $$\begin{aligned} \left. \begin{aligned}&B_1 = 2C_1I_3^{-1/3}+2C_2I_3^{-2/3}I_1\,,\\&B_2 = -2C_2I_3^{-2/3}\, \textrm{and}\\&B_3 = \frac{-2}{3}C_1I_3^{-1/3}I_1-\frac{4}{3}C_2I_3^{-2/3}I_2, \end{aligned} \right. \end{aligned}$$

are terms determined using invariants $I_1$, $I_2$ and $I_3$ of the right Cauchy–Green deformation tensor $\textbf{C}=\textbf{F}\textbf{F}^{\textrm{T}}$ with $\textbf{F}$ being the second-order tensor of the deformation gradient. Constants $\hbox {C}_1$ and $\hbox {C}_2$ are material parameters of the deviatoric part of the Mooney–Rivlin formulation. In addition to the isotropic part, the stress tensor for skeletal muscle tissue also contains an anisotropic part. This is also true for the tendon and aponeurosis. They, however, do not exhibit any active behaviour. Hence, in all generality, the anisotropic second Piola–Kirchhoff tensor $\textbf{S}_\textrm{aniso}$ is expressed as a sum of a passive $\textbf{S}_\textrm{passive}$ and active $\textbf{S}_\textrm{active}$ term, i.e.,

16 $$\begin{aligned} \textbf{S}_{\textrm{aniso}} = \textbf{S}_{\textrm{passive}} + \gamma \,\alpha \,\textbf{S}_{\textrm{active}} \,. \end{aligned}$$

In Eq. (16), the parameter $\alpha$ is related to the overall level of skeletal muscle tissue activity. The following formulations are chosen for the passive and active second Piola–Kirchhoff stresses:

$$\begin{aligned} \left. \begin{aligned}&\nonumber \textbf{S}_\textrm{passive} = {\left\{ \begin{array}{ll} \frac{1}{\Lambda _{\textrm{s}}^2} C_\textrm{3}\left( \Lambda _f^{C_\textrm{4}}-1\right) \textbf{M},\, \text {if}\,\Lambda \ge 1,\\ 0,\, \text {otherwise} \end{array}\right. } \\&\textbf{S}_\textrm{active} = {\left\{ \begin{array}{ll} \frac{\sigma _{\textrm{max}}}{\Lambda ^2_{\textrm{s}}}\text {exp}\left( -\left| {\frac{\left( {\Lambda _{\textrm{s}}}\backslash {\Lambda _{\textrm{opt}}}\right) -1}{\Delta W_\textrm{asc}}}\right| ^{\nu _\textrm{asc}}\right) \textbf{M},\, \text {if}\,\Lambda _{\textrm{s}} \le \Lambda _\textrm{opt},\\ \frac{\sigma _{\textrm{max}}}{\Lambda ^2_{\textrm{s}}}\text {exp}\left( -\left| {\frac{\left( {\Lambda _{\textrm{s}}}\backslash {\Lambda _{\textrm{opt}}}\right) -1}{\Delta W_\textrm{dsc}}}\right| ^{\nu _\textrm{dsc}}\right) \textbf{M},\, \text {otherwise} \end{array}\right. } \end{aligned} \right. \end{aligned}$$

where $C_\textrm{3}$ and $C_\textrm{4}$ are material parameters for the passive components, while $\Delta W_\textrm{asc}$, $\nu _\textrm{asc}$, $\Delta W_\textrm{dsc}$ and $\nu _\textrm{dsc}$ influence the active stress component. $\Lambda _{\textrm{s}}$ is the fibre stretch equivalent to $\sqrt{I_4}$, where $I_4 = \textrm{tr}(\varvec{\textrm{MC}})$ and $\textbf{M}$ being the structural tensor defined by the dyadic product, $\textbf{a}_0 \mathbf {\otimes } \textbf{a}_0$, of the fibre orientation vector $\textbf{a}_0$ as given or computed for the reference configuration. Finally, $\sigma _{\textrm{max}}$ is defined as the maximum achievable active stress at the optimal fibre length $\Lambda _{\textrm{opt}}$. To describe the passive behaviour of the skeletal muscle tissue, tendon, and aponeurosis, we chose constitutive descriptions containing 7 material parameters. Anatomically prevalent pre-stresses play a crucial role in the development of passive and active stresses within a musculoskeletal system (Avci and Röhrle 2024). We model the pre-stress within the *m. rectus femoris* by considering a pre-defined fibre stretch, i.e., applying a realistic and constant stretch value as a load case. More specifically, for a given configuration, the actual stretch of muscle fibres $\Lambda _\textrm{s}$ is determined by:

17 $$\begin{aligned} \Lambda _\textrm{s}=\Lambda _\textrm{ini}+\bar{\Lambda }_\textrm{c}, \end{aligned}$$

where $\Lambda _\textrm{ini}$ represents the initial stretch in muscle fibres, while $\overline{\Lambda }_\textrm{c}$ is determined from the fourth invariant of the right Cauchy–Green deformation tensor, resulting from tissue deformations due to internal loads, muscle contraction, passive stretching or external forces such as body forces, e.g., gravitational effects.

The calibration of the constitutive laws were based on experimental compression (Mohammadkhah et al. 2016) and tensile tests (Ngwangwa and Nemavhola 2021; Ward et al. 2020). The optimum parameters were identified using a least-square fitting algorithm of the simulated response to the experimental data. The parameters are listed in Table 2. The material model has been implemented as a user material subroutine in ANSYS LS-DYNA v12.0^1^ in a previous study by Avci and Röhrle (2024) and is used for all FE simulations here.

#### Load cases for mechanical analysis

The objective of the load cases is to analyse (a) the passive behaviour of *m. rectus femoris* as it would be during mechanical tension tests under in vitro conditions, i.e., without pre-stress effects and (b) the active behaviour of *m. rectus femoris*, i.e., simulating the isometric contraction condition of the muscle within the musculoskeletal system with pre-stress effects. Following mechanical load cases are considered in the current study: First, we consider a passive stretch of the MTA complex. To do so, we fix the upper face of the proximal tendon, i.e., zero displacements at $\bar{T}_\textrm{inlet}$, and apply nodal forces summing up to $\bar{F}_\textrm{2}=5\,$N at the lower face of the distal tendon, i.e., force boundary conditions at $\bar{T}_\textrm{outlet}$. The direction of $\bar{F}_\textrm{2}$ is parallel to axis $\textbf{e}_{\textrm{2}}$. The value of $\bar{F}_\textrm{2}$ is increased linearly from $0\,$N to $5\,$N. The remaining boundary points are not specified. We will refer to this passive stretch simulation as load case 1. Second, we consider an isometric contraction of *m. rectus femoris*. This is referred as load case 2. Load case 2 is done in two load steps, i.e., by LS-2.1 (applying an initial stretch) and LS-2.2 (applying an active contraction), described as follows:

LS-2.1: The initial stretch field is obtained by solving a modification of Eq. (17), i.e.,
  18 $$\begin{aligned} \Lambda _\textrm{s}^{\textrm{ini}}=\Lambda _\textrm{ini}^\textrm{app}+\bar{\Lambda }_\textrm{c}^{\textrm{ini}}, \end{aligned}$$
  with $\Lambda _\textrm{ini}^\textrm{app}$ set to 1.25 and applied to the entire muscle as an internal load. $\bar{\Lambda }_\textrm{c}^{\textrm{ini}}$ is the non-uniform, inhomogeneous stretch field obtained after the application of $\Lambda _\textrm{ini}^\textrm{app}$ under static equilibrium conditions. During this load step, the proximal and distal tendon ends remain fixed. We refer to the study by Avci and Röhrle (2024) for further details on the application and interpretation of initial stretch fields.
LS-2.2: The muscle activation parameter $\alpha$, cf. Eq. (16), is gradually increased from 0 to 1. The aponeurosis and tendons receive no activation signal. Also, the tendon ends remain fixed during this load-step to reproduce the contraction under isometric conditions.

All load cases were computed using an implicit time integration scheme in LS-DYNA, employing a full Newton iterative solver with Broyden–Fletcher–Goldfarb–Shanno (BFGS) updates. The quasi-static FE simulations were executed on a Linux workstation equipped with four Intel(R) Xeon(R) processors, utilising 52 MB of RAM and requiring 35 min of computational time.

## Results

Based on the *m. rectus femoris*, we present results for the fibre tractography, MRI-based modelling of the skeletal muscle geometry, automatic estimation of the anterior aponeurosis including the calculation of its volume (Sect. 3.1), the muscle’s fibre architecture determined by our 3D-thermal (Laplacian)-based simulations (Sect. 3.2), and the structural simulations to highlight and analyse the difference between thermal-simulation-based and DTI-based fibre architectures (Sect. 3.3). The latter is based on FE-simulation of the mechanical behaviour of the *m. rectus femoris* informed by the two different fibre architectures.

### Fibre tractography and determination of the geometry and internal structures of *m. rectus femoris*

#### Fibre tracts from tractography

**Fig. 3:**
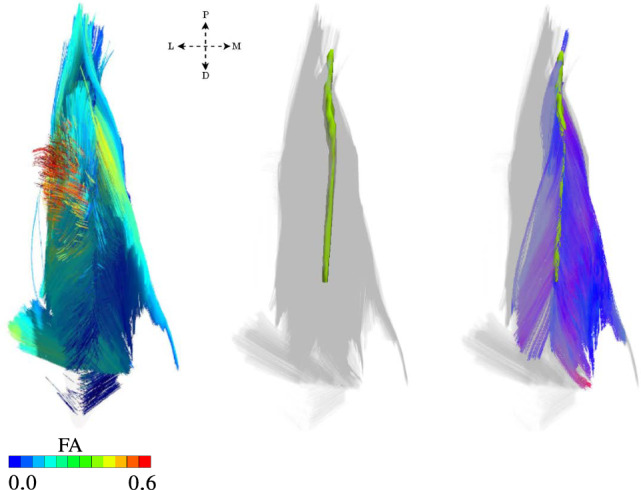
Illustration of estimated muscle fibres and the geometrical representation of the aponeurosis. From left to right: all extracted fibres exhibit a fractional anisotropy, FA, that ranges between 0.0 and 0.6; geometrical representation of the anterior aponeurosis (light green) segmented from MRI data; and fibres obtained from tractography that originate or terminate at the aponeurosis. The letters P, D, L and M refer to anatomical directions proximal, distal, lateral, and medial respectively

Based on the DTI data described in Sect. 2.1 and the parameters for fibre tractography given in Sect. 2.2, we obtained for the bi-pennate *m. rectus femoris* a total of $109\,710$ individual skeletal muscle fibres tracks. The results associated with the fibre tractography, segmentation of the aponeurosis, and fibres originating, terminating or intersecting with the aponeurosis are shown in Fig. 3.

#### Automatic determination of *m. rectus femoris*’ aponeurosis

**Fig. 4:**
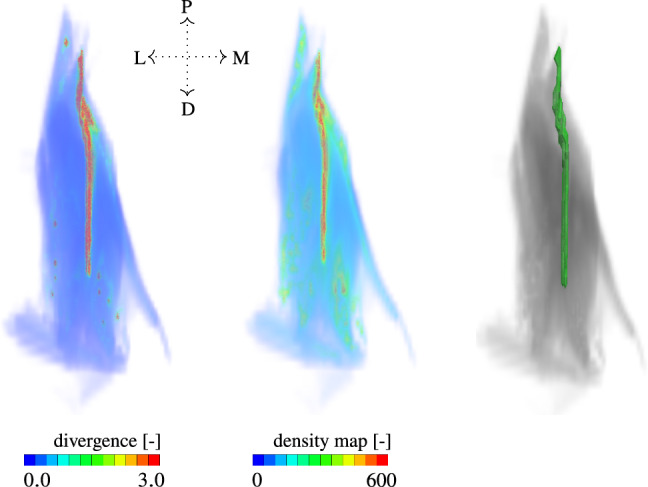
Numerical divergence of fibre orientation field (left), fibre density map for extracted muscle fibres (middle) and the derived aponeurosis volume shown in dark green from the largest connected region exhibiting simultaneously high numerical divergence and fibre density map values, along with the adjoining muscle fibres (right). The letters P, D, L and M refer to anatomical directions proximal, distal, lateral and medial, respectively

Following Sect. (2.2), we depict in Fig. 4 the results of the automatically reconstructed *m. rectus femoris*’ aponeurosis (cf. Fig. 4 (right)). Further, Fig. 4 visualises the respective numerical divergence values, cf. Fig. 4 (left) and the fibre density map, cf. Fig. 4 (middle). The numerical *divergence* values range between 0.0 and 3.0 while fibre density stretches from 0.0 to 600.0.

**Fig. 5:**
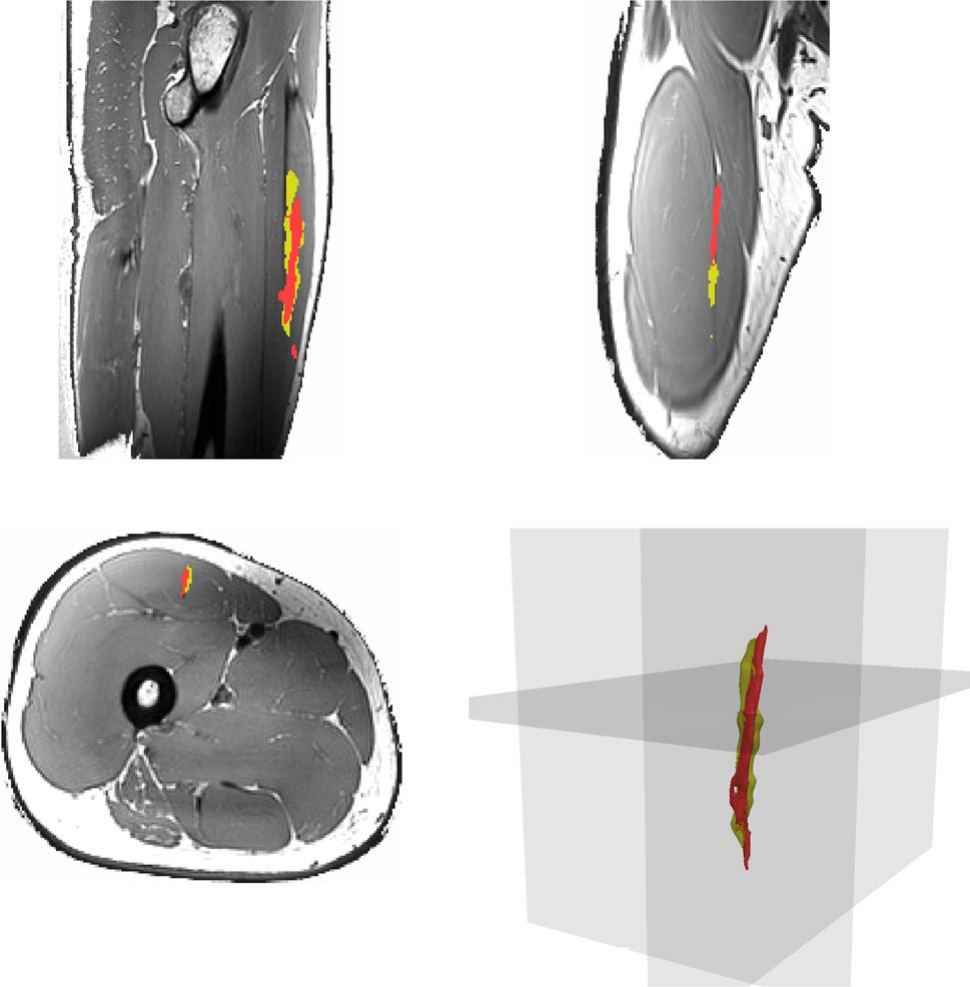
Illustration of manual segmentation of aponeurosis volume $V_{\text {MRI}}^{\text {A}}$, shown in yellow and fibre density based estimation of aponeurosis $V_{\text {DTI}}^{\text {A}}$, shown in red, with coronal (top left), sagittal (top right), axial (bottom left) and 3-D isometric (bottom right) views

Figure 5 illustrates for different anatomical planes the aponeurosis for the manual MRI-based and automatic DTI-based reconstructions. Counting the number of voxels of the two geometrical representations of the same aponeurosis results in 2702 and 2035 voxels for $V_{\text {MRI}}^{\text {A}}$ and $V_{\text {DTI}}^{\text {A}}$ respectively. Further, we compare the volumes of the manually and automatically segmented aponeurosis by computing the Dice Similarity Coefficient, *DSC*, which is defined as

19 $$\begin{aligned} \left. \begin{aligned}&DSC = 2\frac{\left| V_\textrm{DTI}^\textrm{A}\bigcap V_\textrm{MRI}^\textrm{A} \right| }{\left| V_\textrm{DTI}^\textrm{A} \right| + \left| V_\textrm{MRI}^\textrm{A} \right| } . \end{aligned} \right. \end{aligned}$$

Herein, $\bigcap$ represents the intersection of voxel sets $V_\textrm{DTI}^\textrm{A}$ and $V_\textrm{MRI}^\textrm{A}$ and $|\cdot |$ is the total number of voxels in the respective voxel set. In our case, we obtain for the *rectus femoris*’ aponeurosis a *DSC* value of 0.83.

#### CAD- and FE-model of the *rectus femoris* and its aponeurosis

**Fig. 6:**
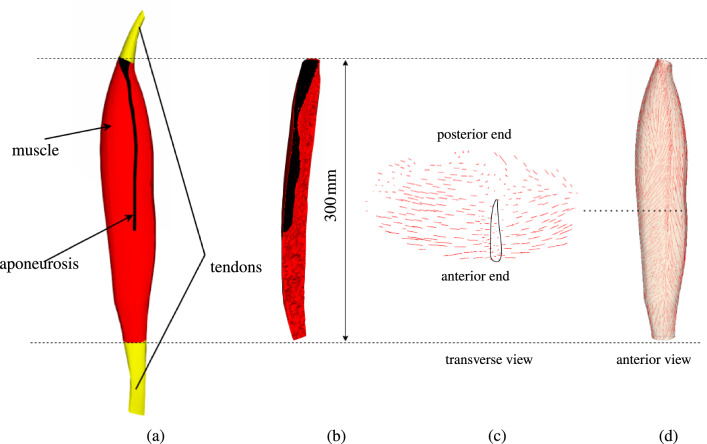
**a** Illustration of the generated CAD-model of *rectus femoris* with its central aponeurosis along with **b** longitudinal cross section of the FE-mesh. **c** Depicted here is a transverse cross section of fibre orientations in the muscle and **d** mapped fibres along with the FE-mesh of the MTA-complex

Volumetric meshing of CAD-surfaces generated by the methodology outline in Sect. (2.2.2) resulted in $347\,212$ tetrahedral elements defined by $65\,604$ node points. The anterior view and the longitudinal cross section of the resulting FE-model are shown in Fig. 6a, b, respectively, whereas in Fig. 6c, d the mapped fibre orientations for a cross-section and looking from anterior on a plane cutting through the muscle are depicted.

The cross section depicts the projection of the fibres in the longitudinal direction as scattered points and those in the transverse direction as horizontal lines. The depicted lines converge towards the aponeurosis (outlined in black), close to the centre at its anterior end. Furthermore, as evident from the anterior view on a plane within the muscle, cf. Fig. 6d, its distal region exhibits several fibres crossing each other at angles close to $90^{\circ }$ and fibres associated with a voxel located close to the outside boundary, especially close to its medial and distal ends, deviate significantly from its neighbouring voxels. This is due to fibres from surrounding tissues, differences in image resolution between MRI and DTI data and signal attenuation caused by shading artefacts (Mirowitz 1999) within the DTI data.

### Determination of *rectus femoris*’ fibre architecture

Our proposed fibre tractography method based on solving the 3D heat equation relies on minimising the averaged cosine distances between a DTI-based fibre tractography and vectors resulting from computing the heat flux in $\bar{\Omega }$. Our optimisation method is based on 200 design points determined by the space-filling algorithm published in Morris and Mitchell (1995). For solving the optimisation problem, we define a metamodel of $\bar{y}_{\bar{\Omega }}$, cf. Eq. (13), in dependence on the optimisation variables $T_1$, $\alpha _1$, and $\alpha _2$. A sensitivity analysis for these input parameters showed that $\alpha _1$ has with 53% the strongest influence on the output response, followed by $\alpha _2$ with 46% of the total percentage of influence on the output response $\bar{y}_{\bar{\Omega }}$. Parameter $T_1$ contributes the least to the total sensitivity. An influence factor of less than 1% was determined. Moreover, determining the accuracy of the metamodel by comparing the predicted values of averaged angular deviations $\bar{y}_{\bar{\Omega }}^{\textrm{pred}}$ from the metamodel and the simulated values of $\bar{y}_{\bar{\Omega }}^{\textrm{sim}}$ from the FE-simulation using the metric Coefficient of Determination, $R^2$, cf. (Coefficient of Determination 2008), resulted in a value of 0.982. This implies that the metamodel approximates $\bar{y}_{\bar{\Omega }}$ within design space $\textbf{D}$ with high accuracy.

**Fig. 7:**
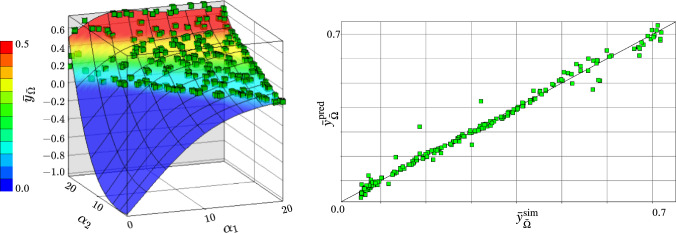
Response surface of $\bar{y}_{\bar{\Omega }}$ with optimization parameters $\alpha _1$ and $\alpha _2$ with contours representing predicted values of averaged angular deviations $\bar{y}_{\bar{\Omega }}^{\textrm{pred}}$ and green points representing simulated values $\bar{y}_{\bar{\Omega }}^{\textrm{sim}}$ (left). The blue region is non-admissible since it is excluded by the optimisation constraint. The adjacent illustration shows the accuracy of the model where $\bar{y}_{\bar{\Omega }}^{\textrm{pred}}$ is plotted against $\bar{y}_{\bar{\Omega }}^{\textrm{sim}}$ (right). Here, the black line represents a perfect $R^2$ score of 1.0

The response surface of the metamodel for output response $\bar{y}_{\bar{\Omega }}$ is shown in Fig. 7 (left), along with the accuracy of the model shown by plotting $\bar{y}_{\bar{\Omega }}^{\textrm{pred}}$ against $\bar{y}_{\bar{\Omega }}^{\textrm{sim}}$ , cf. Fig. 7 (right). From Fig. 7, one clearly observes the high accuracy of the metamodel and the nonlinear behaviour in $\alpha _1$ and $\alpha _2$ of the response surface. The solution of the optimisation problem Eq. (11) results in an optimal set of parameters $T_1^{\textrm{opt}}$, $\alpha _1^{\textrm{opt}}$ and $\alpha _2^{\textrm{opt}}$. They are tabulated in Table 3.

**Table 3:** Set of optimal values of optimization parameters $T_1^{\textrm{opt}}$, $\alpha _1^{\textrm{opt}}$ and $\alpha _2^{\textrm{opt}}$

Parameter	$T_1^{\textrm{opt}}$	$\alpha _1^{\textrm{opt}}$	$\alpha _2^{\textrm{opt}}$
Optimum	− 30.4513	2.2646	2.7554

**Fig. 8:**
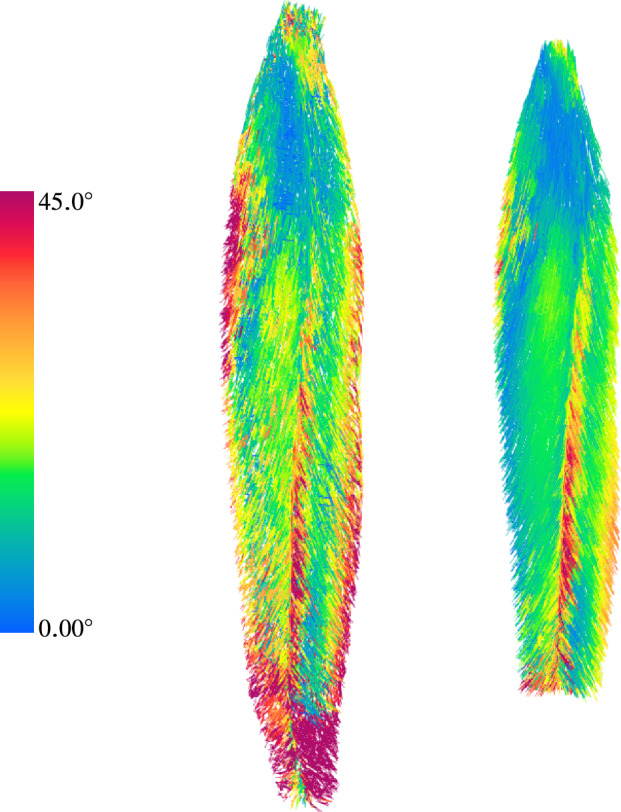
Unit vectors of thermal flux $\textbf{a}_{\textrm{0}}^{\textrm{therm}_{(\textrm{opt})}}$ with contours of $y(\textbf{s}^{\textrm{opt}})$ in entire muscle-aponeurosis-complex with region $\Omega$ (left), and in sub-region $\bar{\Omega }$ (right)

Naturally, the fibre orientation determined by employing the optimal parameters given in Table 3$\textbf{a}_{\textrm{0}}^{\textrm{therm}_{(\textrm{opt})}}$ deviates from the respective one obtained through DTI-based tractography, i.e., $\textbf{a}_{\textrm{0}}^{\textrm{DTI}}$. An illustration of the vector field of the vector field $\textbf{a}_{\textrm{0}}^{\textrm{therm}_{(\textrm{opt})}}$ for the entire MTA-complex $\Omega$ and sub-region $\bar{\Omega }$ is given in Fig. 8. The fibres are coloured based on the absolute values of angular deviation between the two methodologies.

The lateral and medial region of $\bar{\Omega }$ exhibits, for the most part, angular deviations that are less than $30^{\circ }$. Only $14.3\%$ of all elements in $\bar{\Omega }$ exhibit angular deviations greater than $30^{\circ }$. Moreover, except for a few elements in the exterior and distal regions, all were less than $45^{\circ }$. The difference in the number of fibres exhibiting angular deviations larger than $45^{\circ }$ in $\Omega$ and $\bar{\Omega }$ is due to our choice in setting up the optimisation problem, in particular by only optimising over sub-region $\bar{\Omega }$ rather than the entire region, $\Omega$. The smaller region $\bar{\Omega }$ excluded all areas potentially influenced by other structures than the fibres of the *rectus femoris* itself and would misguide any optimisation procedure.

#### Analysis of distribution and mesh sensitivity of angular deviations of thermal simulation-informed fibre model

**Fig. 9:**
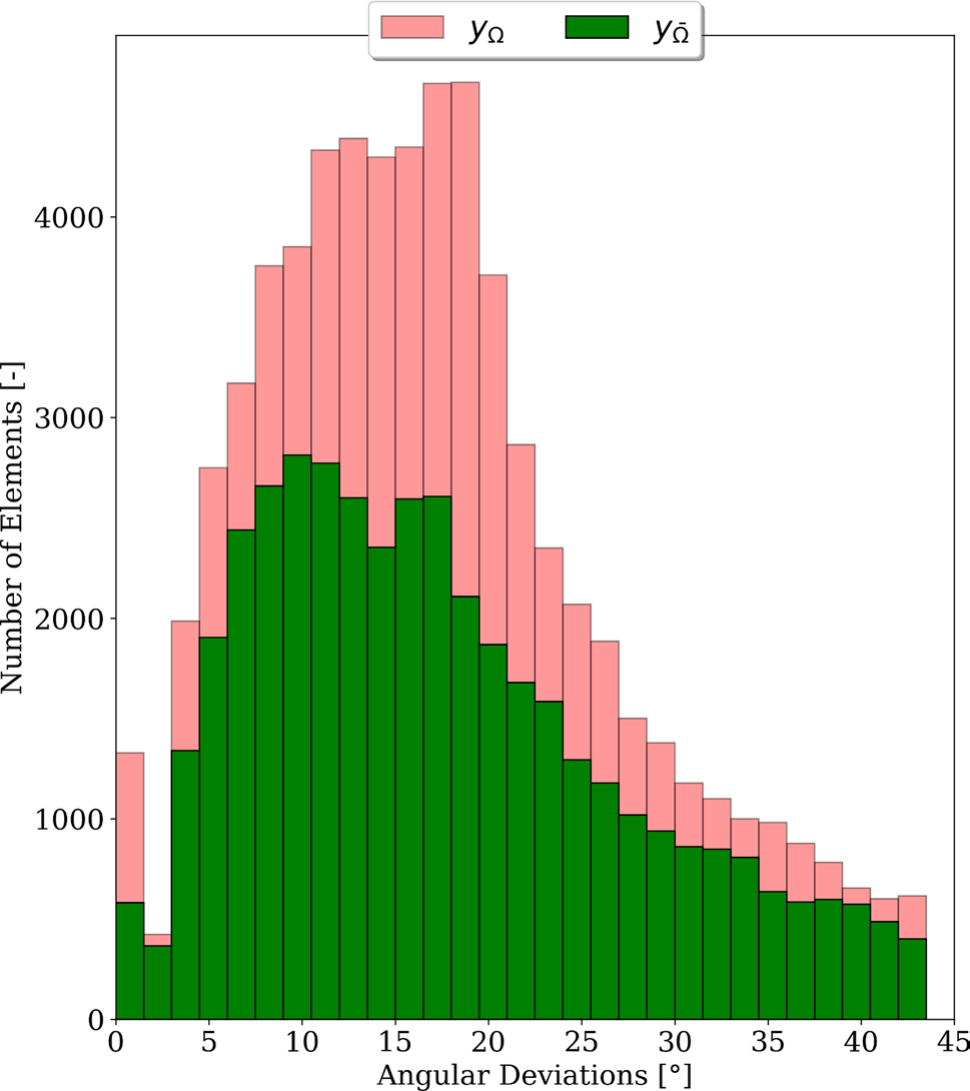
Histograms of angular deviations $y(\textbf{s}^{\textrm{opt}})$ in region $\Omega$ and sub-region $\bar{\Omega }$. It should be noted that the y-axis here is represented using a logarithmic scale

The distribution of angular deviations in region $\Omega$ and sub-region $\bar{\Omega }$ is illustrated as histograms in Fig. 9. Most fibres exhibit angular deviations between $10^{\circ }$ and $20^{\circ }$, whereas lowest values are reported for angular deviations between $2^{\circ }$ and $5^{\circ }$, and beyond $45^{\circ }$. The mean values of angular deviations in sub-region $\bar{\Omega }$ and $\Omega$ were $14.25^\circ \,\pm \,10.36^\circ$ and $14.95^\circ \,\pm \,11.88^\circ$, respectively. The histograms show that angular deviations in both regions have a similar distribution. It also highlights the advantage of excluding the additional elements in $\Omega$ which leads to a lower mean and standard deviation of $y(\textbf{s}^{\textrm{opt}})$ in $\bar{\Omega }$.

**Fig. 10:**
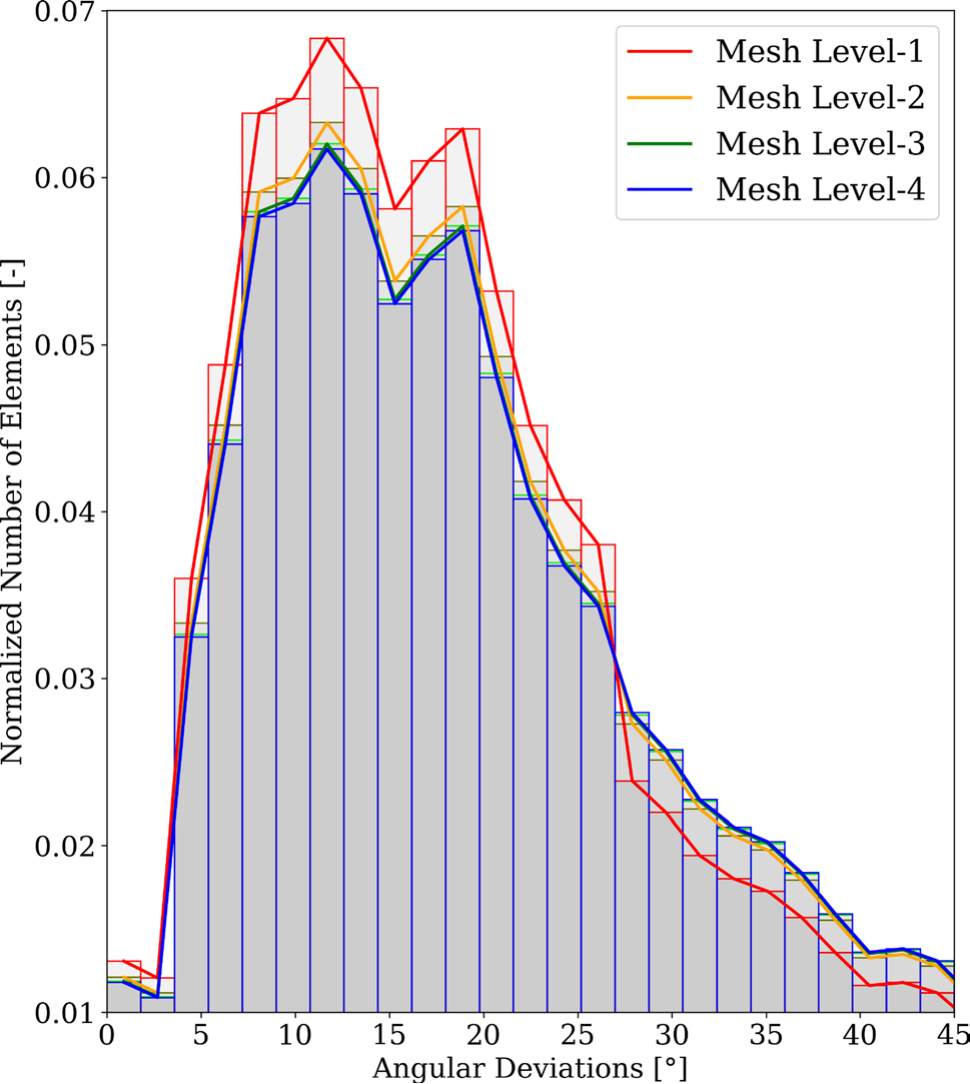
Histograms of angular deviations $y(\textbf{s}^{\textrm{opt}})$ in region $\bar{\Omega }$ for different mesh levels

The mesh sensitivity of thermal flux-based fibre orientations has been studied using varying mesh sizes. We considered four different meshes, termed Mesh Level 1, 2, 3 and 4. They contained $135\,215$, $347\,212$, $2\,134\,872$ and $4\,910\,206$ solid tetrahedral elements, respectively. Angular deviations were computed on $\bar{\Omega }$ for each of these meshes. The histograms, normalised to the total number of elements of each mesh level, are illustrated in Fig. 10. For the coarsest mesh, Mesh Level 1 (in red), slightly higher values for $\bar{y}_{\bar{\Omega }}<25^\circ$ and slightly lower for $\bar{y}_{\bar{\Omega }}>25^\circ$ were observed. This difference arises due to the mapping of $\textbf{a}_{\textrm{0}}^{\textrm{DTI}}$ on a coarse mesh. As the number of elements increases with every mesh level, these differences become significantly smaller and the relative difference between the normalised values is smaller and smaller. Overall, the vector field of fibre orientations $\textbf{a}_{\textrm{0}}^{\textrm{therm}_{(\textrm{opt})}}$ converges. This can also be seen if we only consider the peak values in the total number of angular deviations. The peak for $\bar{y}_{\bar{\Omega }}$ is for all mesh levels close to 10$^\circ$.

### Mechanical analysis of DTI- and thermal simulation-informed fibre architectures

**Fig. 11:**
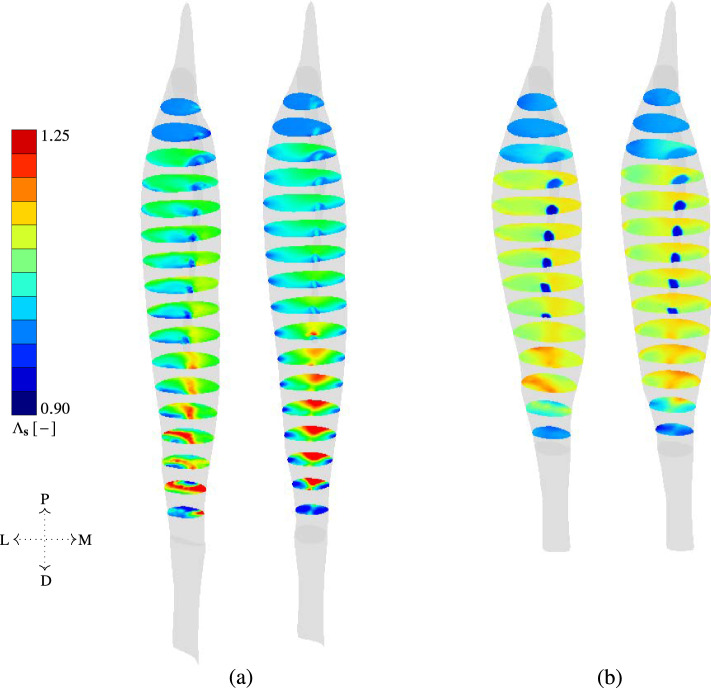
Comparison of fibre stretch $\Lambda _\textbf{s}$ between FE-mesh mapped DTI-informed (left) and thermal simulation-informed fibre orientations (right) for **a** load case 1 (passive stretching) and **b** load case 2 (isometric contraction) using transverse cross-sections defined in the undeformed configuration. The letters P, D, L and M refer to anatomical directions proximal, distal, lateral and medial respectively

To assess the influence of the different fibre orientation fields on the overall mechanical behaviour of skeletal muscle mechanics, we perform a comparative analysis of a FE-model of the MTA-complex once informed with a DTI-based fibre field $\textbf{a}_{\textrm{0}}^{\textrm{DTI}}$, and once with a thermal-simulation-based fibre field $\textbf{a}_{\textrm{0}}^{\textrm{therm}_{(\textrm{opt})}}$. The mechanical behaviour is evaluated by determining both fibre architectures’ stretch level $\Lambda$ in their deformed configuration. The difference in stretch field between the two fibre architectures is illustrated in Fig. 11 using transversal cross sections every $20\,$mm. All cross sections are visualised in the deformed configuration as a result of load case 1 and load case 2.

#### Mechanical analysis of passive stretch of *rectus femoris*

Figure 11a compares the fibre stretch for both models for load case 1, i.e., under passive deformation. The structural model based on $\textbf{a}_{\textrm{0}}^{\textrm{DTI}}$, cf. Fig. 11a (left), leads to discontinuous stretch fields with unequal stretch values on either side of the aponeurosis. Overall, the stretch increases towards the distal end of the muscle. Within the interior of the tissue, the model based on the DTI-based tractography exhibits higher fibre stretch values on the medial side of the aponeurosis than on the lateral side. On the other hand, the muscle deformation of the *rectus femoris* based on $\textbf{a}_{\textrm{0}}^{\textrm{therm}_{(\textrm{opt})}}$, cf. Fig. 11a (right), exhibits a smoother distribution of the fibre stretch. This can be well observed at the external surface of the skeletal muscle tissue, which was excluded from the optimization algorithm due to expected misalignments in the DTI fibre field (cf. Sect. 2.3.2).

The stretch field for the model informed by the thermal-flux-based fibre orientations exhibits similar stretch values on either side of the aponeurosis with $\Lambda$ close to 1.10, extending even beyond the aponeurosis boundary itself. This effect is owed to the choice of applied thermal BCs, resulting in a vector field similar on either side of the aponeurosis. Although a difference in stretch is not obviously visible on either side of the aponeurosis, there are significant differences between the anterior and posterior ends of the muscle for $\textbf{a}_{\textrm{0}}^{\textrm{therm}_{(\textrm{opt})}}$. The posterior-distal end is, for example, a region in which both models predict higher local stretch values.

#### Mechanical analysis of isometric contraction of *rectus femoris*

Similar observations hold for the soft tissue deformations after load case 2, i.e., subject to an isometric contraction. Discontinuous fibre stretch fields occur primarily in the model informed by $\textbf{a}_{\textrm{0}}^{\textrm{DTI}}$, cf. Fig. 11b. The discontinuity in the fibre stretch field is especially dominant in the external surface of the skeletal muscle tissue. Similar to load case 1, different fibre stretch values are observed in the interior regions of the skeletal muscle tissue on either side of the aponeurosis, cf. Fig. 11b (left). Here, stretch values on the lateral side of the aponeurosis range between 1.08 and 1.11, while the medial side exhibits values ranging from 1.14 to 1.18. For the $\textbf{a}_{\textrm{0}}^{\textrm{therm}_{(\textrm{opt})}}$-informed muscle model, the differences in stretch values on either side of the aponeurosis are much smaller, cf. Fig. 11b (right). In fact, similar to load case 1, the posterior-distal end is characterised by local regions of high stretch values, with lower stretch values observed in the DTI-informed FE model than in the $\textbf{a}_{\textrm{0}}^{\textrm{therm}_{(\textrm{opt})}}$-informed one.

#### Analysis of relative inclinations of DTI- and thermal simulation-informed fibre orientations

The difference in the deformation behaviour of the same model informed by the two different fibre architectures is attributed to the angular deviations inherently observed in the $\textbf{a}_{\textrm{0}}^{\textrm{therm}_{(\textrm{opt})}}$ and $\textbf{a}_{\textrm{0}}^{\textrm{DTI}}$ fibre field (cf. Fig. 8). Since skeletal muscle fibres are assumed in our models to be the primary tensile load-bearing structures, we analysed the steepness of fibre directions relative to the axis of external loading. However, fibre orientations in our models do not inherently have a defined direction. Therefore, we first aligned the two fibre orientations to the primary axis of the muscle $\textbf{e}_2$ such that $\textbf{a}_{\textrm{0}}^{\textrm{therm}_{(\textrm{opt})}} \cdot \textbf{e}_2 \ge 0$ and $\textbf{a}_{\textrm{0}}^{\textrm{DTI}} \cdot \textbf{e}_2 \ge 0$. We computed the relative fibre inclinations between the two fields of fibre orientations $\widetilde{\alpha }_0$ using the following relationship:

20 $$\begin{aligned} \begin{aligned}&\widetilde{\alpha }_0^{(k)} = \frac{\pi }{2} -\arccos \left( \left( \frac{\textbf{a}_{\textrm{0}}^{\textrm{DTI,k}}- \textbf{a}_{\textrm{0}}^{\textrm{therm,k}}(\mathbf {s^{\textrm{opt}}})}{\Vert \textbf{a}_{\textrm{0}}^{\textrm{DTI,k}}-\textbf{a}_{\textrm{0}}^{\textrm{therm,k}}(\mathbf {s^{\textrm{opt}}})\Vert }\right) \cdot \textbf{e}_2\right) ,\\ \end{aligned} \end{aligned}$$

for all *k* with *k* referencing a FE-mesh element.

**Fig. 12:**
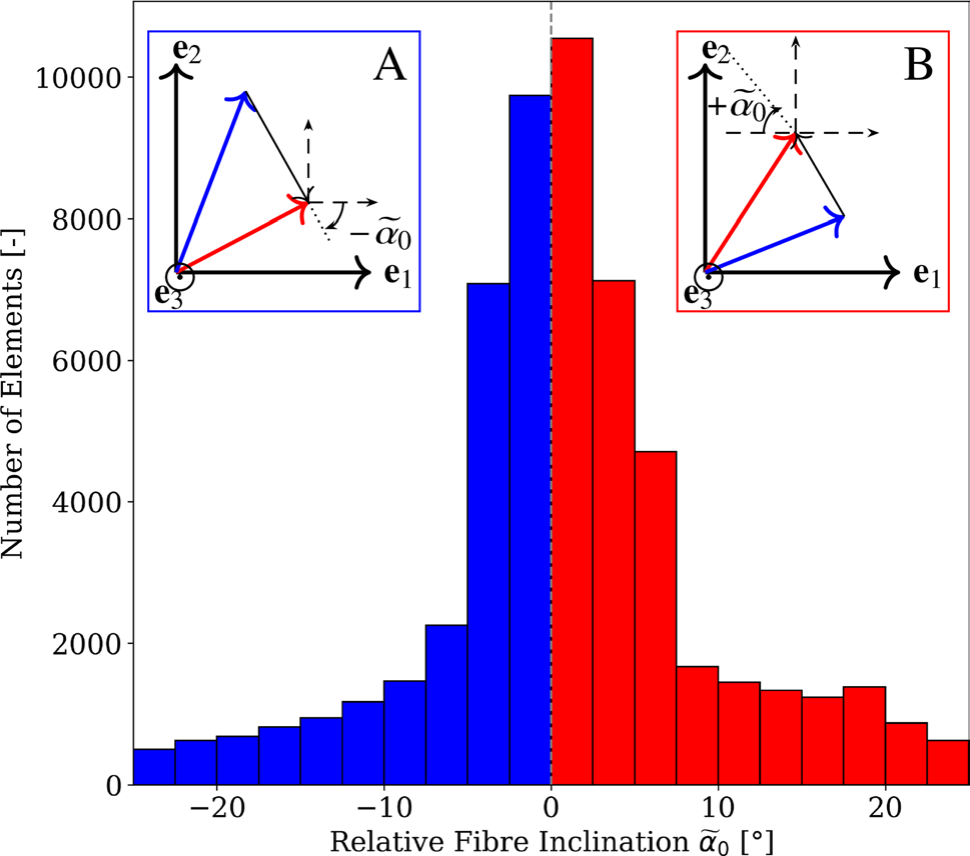
Histograms of relative fibre inclination $\widetilde{\alpha }_0$ with red bars representing higher and blue bars representing lower inclinations of $\textbf{a}_{\textrm{0}}^{\textrm{DTI}}$ relative to $\textbf{a}_{\textrm{0}}^{\textrm{therm}_{(\textrm{opt})}}$. Box A depicts a scenario of blue bars where $\textbf{a}_{\textrm{0}}^{\textrm{DTI}}$ (red arrow) is flatter relative to $\textbf{a}_{\textrm{0}}^{\textrm{therm}_{(\textrm{opt})}}$ (blue arrow) and box B illustrates a case of red bars where $\textbf{a}_{\textrm{0}}^{\textrm{DTI}}$ (red arrow) is steeper compared to $\textbf{a}_{\textrm{0}}^{\textrm{therm}_{(\textrm{opt})}}$ (blue arrow)

The scalar field of fibre inclination $\widetilde{\alpha }_0$ is computed in the undeformed configuration and is depicted as histograms with a logarithmic scale in Fig. 12. Here, positive bars (in red) indicate a higher steepness of $\textbf{a}_{\textrm{0}}^{\textrm{DTI}}$, and negative bars (in blue) imply flatness of $\textbf{a}_{\textrm{0}}^{\textrm{DTI}}$ relative to $\textbf{a}_{\textrm{0}}^{\textrm{therm}_{(\textrm{opt})}}$. The peak of the histograms of $\widetilde{\alpha }_0$ is observed close to $0^{\circ }$, which indicates close similarity in relative inclinations of the two fibre architectures. Close to 5000 elements exhibit $\widetilde{\alpha }_0>+20^\circ$, and fewer than 2500 elements have $\widetilde{\alpha }_0<-10^\circ$, which constitutes less than $3\%$ of the total elements in the FE-mesh for both fibre architectures. Additionally, for $0^\circ<\widetilde{\alpha }_0<2.5^\circ$ and $\widetilde{\alpha }_0>10.0^\circ$, the number of elements with steeper relative inclination for $\textbf{a}_{\textrm{0}}^{\textrm{DTI}}$ is higher than that for the model informed by $\textbf{a}_{\textrm{0}}^{\textrm{therm}_{(\textrm{opt})}}$. The mean and standard deviation of $\widetilde{\alpha }_0$ are $0.44^\circ \,\pm \,4.48^\circ$.

**Fig. 13:**
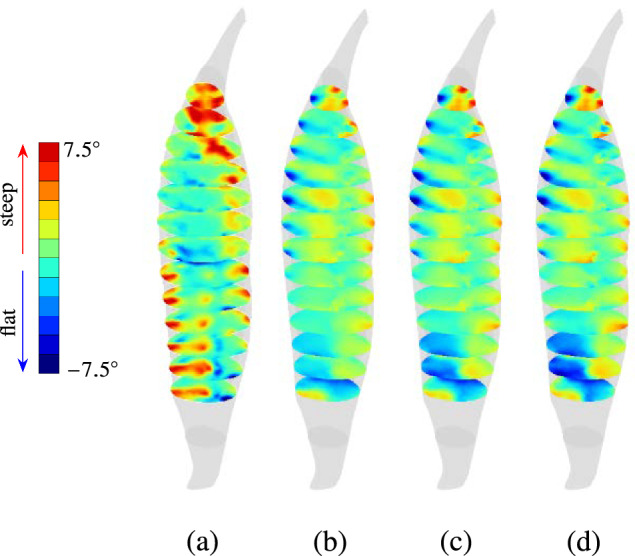
Contours of relative fibre inclination for load case 1 expressed in degrees for **a** undeformed fibre orientation $\widetilde{\alpha }_0$ and deformed fibre orientation $\widetilde{\alpha }$ at **b** $30\%$, **c** $60\%$, **d** $90\%$ of maximum value of $\bar{F}_{\textrm{2}}$

The computation of relative fibre inclination $\widetilde{\alpha }_0$ enables a direct comparison of both the fibre architectures with a single scalar field, i.e., scalar field of $\widetilde{\alpha }_0$. The spatial distribution of $\widetilde{\alpha }_0$ is illustrated in Fig. 13a by visualising a transversal cross section of the undeformed configuration for every $20\,\text {mm}$. Here, one observes that the belly region exhibits similar $\widetilde{\alpha }_0$ values in the interior regions of the skeletal muscle. At the lateral and medial ends of the muscle belly, steeper angular inclinations are observed for the $\textbf{a}_{\textrm{0}}^{\textrm{DTI}}$-informed model. Similar to the results of fibre stretch in load cases 1 and 2 (cf. Fig. 11), values of $\widetilde{\alpha }_0$ differ in the muscle belly between the medial and lateral ends, with $\textbf{a}_{\textrm{0}}^{\textrm{DTI}}$ exhibiting steeper relative inclinations on the lateral side and flatter orientations on the medial side. As one approaches the distal-most cross-section, the distribution of $\widetilde{\alpha }_0$ becomes discontinuous, with $\textbf{a}_{\textrm{0}}^{\textrm{DTI}}$ exhibiting significantly flatter inclination angles towards the anterior and medial ends, while flatter inclination angles are observed for the $\textbf{a}_{\textrm{0}}^{\textrm{therm}_{(\textrm{opt})}}$-informed model on the lateral side.

In the deformed state, the relative fibre inclination values change significantly. In load case 1, the muscle deforms passively under the action of external force $\bar{F}_\textrm{2}$ (cf. Sect. 2.4.2). As the value of $\bar{F}_{\textrm{2}}$ increases, the passive deformation of the muscle results in a new distribution of relative inclination angles. In the deformed configuration, relative fibre inclination $\widetilde{\alpha }$ is computed by replacing in Eq. (20) the initial orientation field $\textbf{a}_{\textrm{0}}$ with $\textbf{F}\,\textbf{a}_{\textrm{0}}$. Here, $\textbf{F}$ represents the deformation gradient for a given deformed state. The change in $\widetilde{\alpha }$ with increase in applied load $\bar{F}_{\textrm{2}}$ is shown in Fig. 13b–d using transversal cross-sections defined in the undeformed configuration. At lower values, i.e., at $30\%$ of maximum applied load $\bar{F}_{\textrm{2}}^{\textrm{max}}=5$N, the inclinations of the two architectures tend to become equal, cf. Fig. 13b. At this value of $\bar{F}_{\textrm{2}}$, a significant reduction in the range of values of $\widetilde{\alpha }$ is observed, i.e., it drops from $-20^\circ$ to $+20^\circ$ ($\widetilde{\alpha }_0$) to a range smaller than $-5^\circ$ to $+5^\circ$.

Upon further increase in $\bar{F}_{\textrm{2}}$, $\widetilde{\alpha }$ no longer significantly changes in the muscle belly region. However, in the distal region of the muscle, the fibre architecture flattens out in the $\textbf{a}_{\textrm{0}}^{\textrm{DTI}}$-informed model. This is observed by the regions coloured blue in the distal region of the muscle in Fig. 13c, d that indicate a flatter inclination of $\textbf{a}_{\textrm{0}}^{\textrm{DTI}}$ compared to $\textbf{a}_{\textrm{0}}^{\textrm{therm}_{(\textrm{opt})}}$. Here, $\widetilde{\alpha }$ is shown at $60\%$ of $\bar{F}_{\textrm{2}}^{\textrm{max}}$, i.e., $\bar{F}_{\textrm{2}}=3.0$N (cf. Fig. 13c) and $90\%$ of maximum $\bar{F}_{\textrm{2}}^{\textrm{max}}$, i.e., $\bar{F}_{\textrm{2}}=4.5$N (cf. Fig. 13d).

The mean and standard values of $\widetilde{\alpha }$ at $30\%$, $60\%$, and $90\%$ of $\bar{F}_{\textrm{2}}^{\textrm{max}}$ value are $0.49^\circ \,\pm \,2.24^\circ$, $0.51^\circ \,\pm \,2.42^\circ$, and $0.49^\circ \,\pm \,2.57^\circ$, respectively. The results indicate a drop in standard deviation of $\widetilde{\alpha }$ at $30\%$ $\bar{F}_{\textrm{2}}^{\textrm{max}}$ ($0.49^\circ \,\pm \,2.24^\circ$) from the standard deviation of $\widetilde{\alpha }_0$ ($0.44^\circ \,\pm \,4.48^\circ$), i.e., in the undeformed configuration. This drop in standard deviation suggests that the fibre inclination of the two fibre architectures tends to become very similar at $30\%$ of $\bar{F}_{\textrm{2}}^{\textrm{max}}$. However, beyond this value of applied force, the muscle stretches further, and the standard deviation of $\widetilde{\alpha }$ increases from $2.24^\circ$ at $30\%$ of $\bar{F}_{\textrm{2}}^{\textrm{max}}$ to $2.42^\circ$ at $60\%$ of $\bar{F}_{\textrm{2}}^{\textrm{max}}$ and $2.57^\circ$ at $90\%$ of $\bar{F}_{\textrm{2}}^{\textrm{max}}$. The increase in standard deviation of $\widetilde{\alpha }$ from low to high values of $\bar{F}_{\textrm{2}}$ indicates an increase in dissimilarity of the two architectures in their deformed configurations at the corresponding values of $\bar{F}_{\textrm{2}}$.

**Fig. 14:**
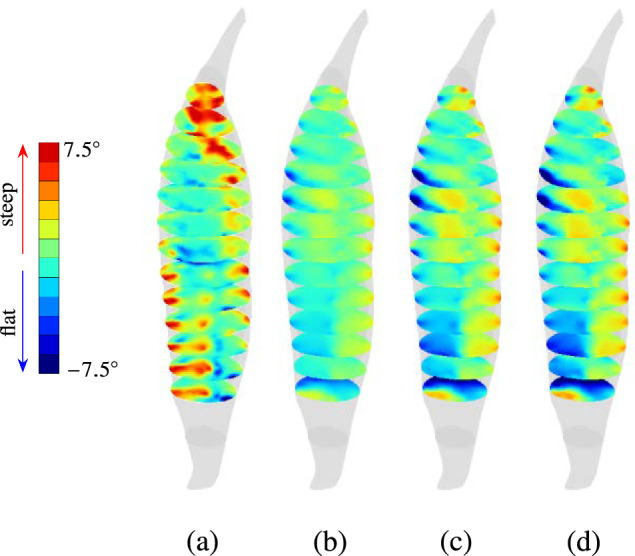
Contours of relative fibre inclination for LS-2.1 expressed in degrees for **a** undeformed fibre orientation $\widetilde{\alpha }_0$ and deformed fibre orientation $\widetilde{\alpha }$ at **b** $\Lambda _\textrm{ini}^\textrm{app}=1.10$, **c** $\Lambda _\textrm{ini}^\textrm{app}=1.25$, **d** $\Lambda _\textrm{ini}^\textrm{app}=1.35$

The relative fibre inclinations of the two fibre architectures also change during deformation of the muscle in isometric contraction, i.e., in load case 2. Here, the mechanical behaviour of both the models are influenced by the initial stretch of fibres $\Lambda _\textrm{s}^{\textrm{ini}}$ (cf. Eq. (18)) applied during LS-2.1. This results in a passive deformation of the muscle and a change in fibre orientation of both the models. The extent of passive deformation depends on the value of $\Lambda _\textrm{ini}^\textrm{app}$ applied during this load step, i.e., higher passive deformation of the muscle at higher values of $\Lambda _\textrm{ini}^\textrm{app}$ and vice versa. Therefore, we analysed the contraction behaviour of both fibre models at various values of $\Lambda _\textrm{ini}^\textrm{app}$, i.e., $\Lambda _\textrm{ini}^\textrm{app}=1.10$, $\Lambda _\textrm{ini}^\textrm{app}=1.25$, and $\Lambda _\textrm{ini}^\textrm{app}=1.35$, and computed the new relative fibre inclination $\widetilde{\alpha }$ in the resulting deformed configuration after application of initial fibre pre-stretch.

A significant change is observed between $\widetilde{\alpha }_0$ in the undeformed configuration, cf. Fig. 14a and $\widetilde{\alpha }$ at $\Lambda _\textrm{ini}^\textrm{app}=1.10$. For this value of $\Lambda _\textrm{ini}^\textrm{app}$, $\widetilde{\alpha }$ values vary between $0^\circ$ and $2^\circ$ in the muscle belly. At the extreme lateral and distal ends of the muscle, $\widetilde{\alpha }$ values close to $-7.5^\circ$ are observed. This is illustrated in Fig. 14b by visualising $\widetilde{\alpha }$ in a transversal cross section of the undeformed configuration for every $20\,\text {mm}$. In Fig. 14c, d, $\widetilde{\alpha }$ is shown for $\Lambda _\textrm{ini}^\textrm{app}=1.25$ and $\Lambda _\textrm{ini}^\textrm{app}=1.35$, respectively.

Compared to the scalar field of $\widetilde{\alpha }$ at $\Lambda _\textrm{ini}^\textrm{app}=1.10$, at higher values of $\Lambda _\textrm{ini}^\textrm{app}$, fibres of the $\textbf{a}_{\textrm{0}}^{\textrm{DTI}}$-informed model get steeper in the muscle belly, resulting in higher active force generation during isometric contraction. Simultaneously, fibres in this model flatten out towards the distal end, allowing for increased shortening of muscle fibres in the region. The mean and standard values of $\widetilde{\alpha }$ at $\Lambda _\textrm{ini}^\textrm{app}=1.10$ are $0.19^\circ \,\pm \,1.23^\circ$. For $\Lambda _\textrm{ini}^\textrm{app}=1.25$ and $\Lambda _\textrm{ini}^\textrm{app}=1.35$, these values are $0.32^\circ \,\pm \,1.94^\circ$ and $0.36^\circ \,\pm \,2.14^\circ$, respectively. Compared to the standard deviation of $\widetilde{\alpha }_0$ ($0.44^\circ \,\pm \,4.48^\circ$) in the undeformed configuration, the standard deviation of $\widetilde{\alpha }$ at $\Lambda _\textrm{ini}^\textrm{app}=1.10$ ($0.19^\circ \,\pm \,1.23^\circ$) exhibits a significant reduction and implies that the inclination of the two architectures is very similar at $\Lambda _\textrm{ini}^\textrm{app}=1.10$. For higher values of $\Lambda _\textrm{ini}^\textrm{app}$, the standard deviation values are higher, i.e., $1.94^\circ$ at $\Lambda _\textrm{ini}^\textrm{app}=1.25$ and $2.14^\circ$ at $\Lambda _\textrm{ini}^\textrm{app}=1.35$ compared to $1.23^\circ$ at $\Lambda _\textrm{ini}^\textrm{app}=1.10$. This indicates that after an initial reduction in standard deviation values at $\Lambda _\textrm{ini}^\textrm{app}=1.10$, the fibre inclinations of both models become increasingly dissimilar at values of $\Lambda _\textrm{ini}^\textrm{app}$ greater than 1.10.

#### Structural response of DTI- and thermal simulation-informed fibre orientations to passive and active loads

**Fig. 15:**
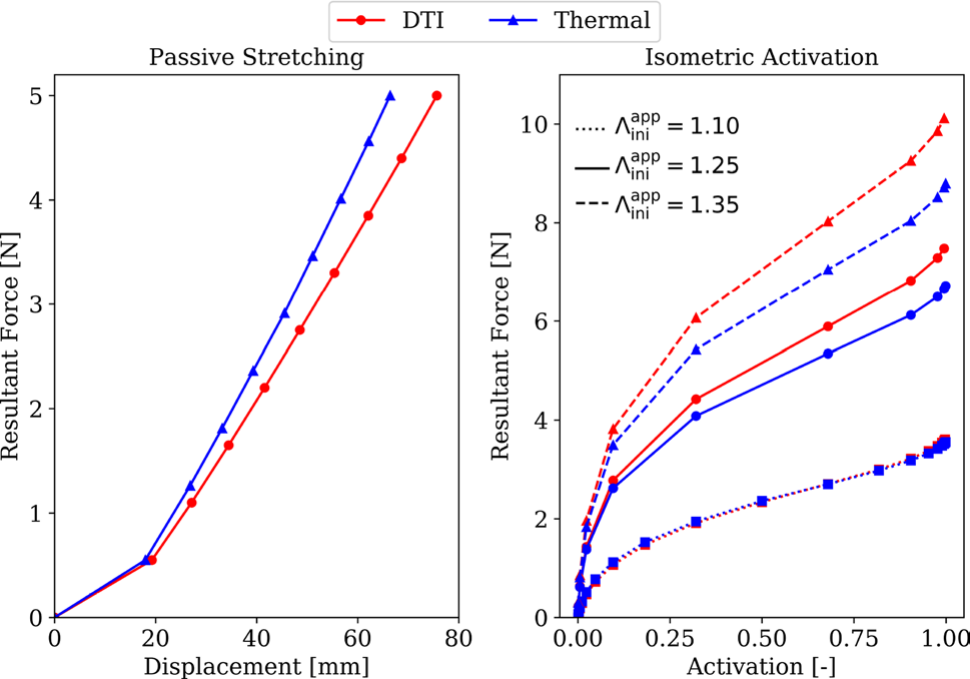
Reaction forces at proximal tendon end for passive stretching (left) and isometric activation (right) for mechanical study of the DTI-informed and thermal simulation-informed fibre models

We attribute the overall structural response of both the models to their relative fibre inclination in the undeformed and deformed configurations. For load case 1, we determine the force-displacement characteristics of both the models by plotting the average displacement of all nodes at boundary $\bar{T}_\textrm{outlet}$ (cf. Fig. 1) against the applied force $\bar{F}_{\textrm{2}}$. This is illustrated in Fig. 15 (left). It is observed here that for values of $\bar{F}_{\textrm{2}}$ below 0.6N, the stiffness of both architectures is similar, which results in nearly equal displacements of the distal tendon. However, as the value of $\bar{F}_{\textrm{2}}$ increases, the flatness of $\textbf{a}_{\textrm{0}}^{\textrm{DTI}}$ (cf. Fig. 13b–d) compared to $\textbf{a}_{\textrm{0}}^{\textrm{therm}_{(\textrm{opt})}}$, especially in the distal region, results in lower stiffness of the $\textbf{a}_{\textrm{0}}^{\textrm{DTI}}$-informed model. This leads to a larger average displacement of the distal end for the $\textbf{a}_{\textrm{0}}^{\textrm{DTI}}$-informed model. Consequently, for this model, a 14% higher displacement of the distal tendon is observed at $\bar{F}_{\textrm{2}}=5\,\text {N}$ compared to the $\textbf{a}_{\textrm{0}}^{\textrm{therm}_{(\textrm{opt})}}$-informed model.

For load case 2, we determine the reaction forces at boundary $\bar{T}_\textrm{inlet}$ (cf. Fig. 1) during isometric contraction of both the models. In Fig. 15 (right), the reaction forces of both models are plotted against the activation level $\alpha \in [0,1]$ of the muscle, applied during LS 2.2 (cf. Sect. 2.4.2) at different values of $\Lambda _\textrm{ini}^\textrm{app}$, i.e., $\Lambda _\textrm{ini}^\textrm{app}=1.10$, $\Lambda _\textrm{ini}^\textrm{app}=1.25$, and $\Lambda _\textrm{ini}^\textrm{app}=1.35$. Here, the two models generate almost identical active forces at $\Lambda _\textrm{ini}^\textrm{app}=1.10$. We attribute this behaviour to the reduction in standard deviation of $\widetilde{\alpha }$ ($0.19^\circ \,\pm \,1.23^\circ$) at $\Lambda _\textrm{ini}^\textrm{app}=1.10$ from its undeformed configuration $\widetilde{\alpha }_0$ ($0.44^\circ \,\pm \,4.48^\circ$). At $\Lambda _\textrm{ini}^\textrm{app}=1.25$ and $\Lambda _\textrm{ini}^\textrm{app}=1.35$, the $\textbf{a}_{\textrm{0}}^{\textrm{DTI}}$-informed model generates $11\%$ and $15\%$ higher forces compared to the $\textbf{a}_{\textrm{0}}^{\textrm{therm}_{(\textrm{opt})}}$-informed model at $\Lambda _\textrm{ini}^\textrm{app}=1.10$. This behaviour is caused by the steeper fibre inclinations in the muscle belly of the $\textbf{a}_{\textrm{0}}^{\textrm{DTI}}$-informed model at these values of $\Lambda _\textrm{ini}^\textrm{app}$ (cf. Fig. 14b–d), which produce higher active forces and result in greater reaction forces compared to the $\textbf{a}_{\textrm{0}}^{\textrm{therm}_{(\textrm{opt})}}$-informed model.

#### Structural response of additional metamodel design points with varying averaged angular deviations

In addition to the optimal set of parameters (cf. Table 3), we computed load cases 1 and 2 for additional designs from the metamodel. The purpose of this analysis is to demonstrate the variation of structural responses with respect to design points that are scattered across the metamodel. The design points are selected from the response surface (cf. Fig. 7) in such a way that the values of averaged angular deviations $\bar{y}_{\bar{\Omega }}$ between 0.0 and 0.5 are covered. This is achieved by picking design points perpendicular to the optimisation constraint, i.e., $\alpha _2 - \alpha _1> 0$. The values of these design points are provided in Table 4.

**Table 4:** Set of additional designs from the metamodel with parameters $T_1^{\textrm{opt}}$, $\alpha _1^{\textrm{opt}}$, and $\alpha _2^{\textrm{opt}}$

Parameter	$T_1^{\textrm{opt}}$	$\alpha _1^{\textrm{opt}}$	$\alpha _2^{\textrm{opt}}$
DP 188	− 2.3952	5.08327	6.1338
DP 107	− 34.4356	10.1851	16.4518
DP 100	− 182.142	6.3018	17.6835
DP 130	− 9.2908	2.9518	16.9341

**Fig. 16:**
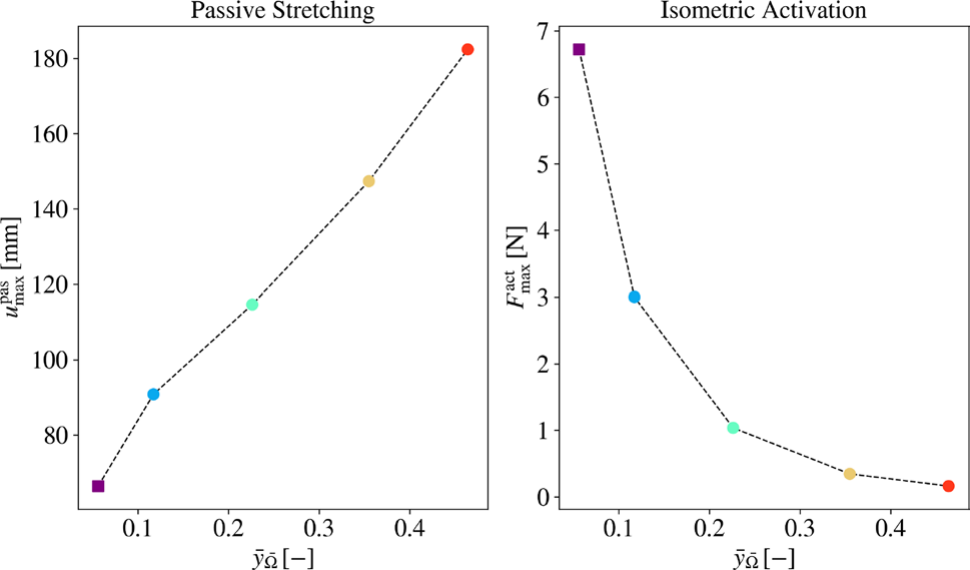
Variation of $u_{\textrm{max}}^{\textrm{pas}}$ (left) and $F_{\textrm{max}}^{\textrm{act}}$ (right) for optimum and additional set of designs from the metamodel. The optimum design point is marked as a square and coloured purple. DP 188, DP 107, DP 100, and DP 130 are marked as circles and coloured light blue, light green, yellow, and red, respectively

We determine the value of average displacement of nodes $u_{\textrm{max}}^{\textrm{pas}}$ at boundary $\bar{T}_\textrm{outlet}$ at the maximum applied force, i.e., $\bar{F}_{\textrm{2}}=5.0\,$N for these designs in load case 1. Additionally, we compute the value of reaction forces $F_{\textrm{max}}^{\textrm{act}}$ for initial fibre pre-stretch $\Lambda _\textrm{ini}^\textrm{app}=1.25$ at $\alpha =1.0$ in LS-2.2. Together with the optimum design, we illustrate the distribution of $u_{\textrm{max}}^{\textrm{pas}}$ and $F_{\textrm{max}}^{\textrm{act}}$ for the corresponding values of $\bar{y}_{\bar{\Omega }}$ in Fig. 16 (left) and (right), respectively. The additional design points are marked as circles, and their colours correspond to the contour of $\bar{y}_{\bar{\Omega }}$ in Fig. 7. The optimum design point is marked as a square and shown in purple.

In load case 1, the thermal simulation-informed designs DP 188, DP 107, DP 100, and DP 130 exhibit $37\%, 72\%, 122\%$, and $175\%$ higher values of $u_{\textrm{max}}^{\textrm{pas}}$ compared to the optimum design. Conversely, in load case 2, these designs generate $55\%, 85\%, 95\%,$ and $98\%$ lower active forces $F_{\textrm{max}}^{\textrm{act}}$ compared to the optimum design. We attribute this to the corresponding values of $\bar{y}_{\bar{\Omega }}$. For instance, DP 188 has a value of averaged angular deviations $\bar{y}_{\bar{\Omega }}=0.11$. This design is close to the optimisation constraint, i.e., $\alpha _2 - \alpha _1> 0$, compared to DP 130, which has a value of $\bar{y}_{\bar{\Omega }}=0.46$. As the distance of the design points from the constraint increases, we observe a decrease in steepness of the thermal simulation-based fibre orientations. Since a steeper fibre orientation field results in lower passive deformation during load case 1, the designs with lower values of $\bar{y}_{\bar{\Omega }}$ exhibit higher values of $u_{\textrm{max}}^{\textrm{pas}}$, resulting in a near-linear relationship between $\bar{y}_{\bar{\Omega }}$ and $u_{\textrm{max}}^{\textrm{pas}}$. In contrast, a steeper fibre orientation field generates low active forces during load case 2. This results in a nonlinear relationship between $\bar{y}_{\bar{\Omega }}$ and $F_{\textrm{max}}^{\textrm{act}}$, where $F_{\textrm{max}}^{\textrm{act}}$ decreases with increasing $\bar{y}_{\bar{\Omega }}$.

## Discussion

The study proposed a semi-automatic method of estimating the geometrical representation of a bi-pennate muscle’s aponeurosis, specifically that of the *m. rectus femoris*, using mathematical operations such as the discrete *divergence* operator and *fibre density map*. The application of this approach is not restricted to the *m. rectus femoris* and can easily be extended to other multi-pennate muscles such as *triceps brachii*. The methodology leverages the geometrical characteristics of bi-pennate muscles with fibre orientations converging at the aponeurosis, resulting in a potential source or sink of the vector field. Furthermore, the number of unique fibres attached at the aponeurosis is significantly greater. Compared to the gold standard of manually segmenting the aponeurosis from high-resolution MR images, we achieved an excellent dice coefficient *DSC* of 0.83. Although we took utmost care in manually segmenting the high-resolution MR images, slight inaccuracies are possible. Variations in joint angles during image acquisition and external body forces, including gravitation, may result in passive deformation of individual muscles, altering the configuration of fibre orientations. Additionally, irregularities in DTI sequences, such as infiltration of fat tissues in muscle or high signal attenuation, affect the vector field of fibre directions obtained by tractography, potentially impacting the DSC. To achieve the best possible outcome using this method, we recommend acquiring DTI data at its highest feasible resolution, given the temporal and spatial constraints. Furthermore, we suggest limiting the computation of aponeurosis identification to a precisely defined RoI.

In this study, we introduced a thermal simulation-based approach for approximating subject-specific fibre orientations in the *rectus femoris* muscle. The methodology includes a metamodel-based optimization procedure for determining BCs in a 3D thermal simulation to generate a heat flux-based fibre architecture by minimizing the differences in averaged angular deviations $\bar{y}_{\bar{\Omega }}$ between DTI-based fibre tractography and thermal simulation-based fibre orientations. In a related study to determine fibre orientations obtained from Laplacian flow, Varvik et al. (2021) reported large angular deviations between fibre orientations obtained from a CFD simulation and DTI-based fibre angles, with the average median and standard deviation of angular deviations being $56.55^{\circ }$ and $17.23^{\circ }$. In contrast, our study demonstrated that Laplacian flow-based fibre directions obtained from thermal simulations can produce accurate results with averaged angular deviations of $14.25^\circ \,\pm \,10.36^\circ$, using optimized values of BCs in a thermal simulation. Additionally, we illustrated the advantage of mesh independence in determining fibre orientations using thermal simulations over CFD-based methods (Handsfield et al. 2017).

Further, we performed a structural analysis to investigate the impact of variations in the underlying muscle fibre field on the resulting deformation of the *m. rectus femoris* muscle. We compared the stretch field of the same skeletal muscle FE-model informed by two different fibre orientations, i.e., from DTI-based fibre tractography and thermal simulation-based fibre orientations, during loading conditions of passive stretching and isometric contraction of the muscle. Notably, the comparison of the stretch field reveals an inhomogeneity of fibre orientations arising from DTI, which is challenging to reproduce in fine detail through Laplacian or Stokes’ field-based approximations. Conversely, the thermal simulation-informed fibre model exhibited continuous and smooth fibre directions, leading to a varying yet homogeneous distribution of fibre stretch during passive stretching and isometric contraction.

Observations from other studies have similarly revealed a varying stretch field within muscles under comparable loading conditions. Rehorn and Blemker (2010) computed a nonlinear distribution of the stretch field in the *biceps femoris* muscle, decreasing from 2.2 in the proximal region to 1.0 in the distal region, while Varvik et al. (2021) demonstrated an inhomogeneous field of first principal strain in the *m. gastrocnemius* muscle with both DTI-based and Stokes flow-based fibre orientations. Furthermore, we provided an explanation of the differences in mechanical responses between the DTI-based model and our thermal simulation-informed model by computing the relative fibre inclinations $\widetilde{\alpha }_0$. By analysing the initial reduction in the standard deviation of relative fibre inclinations from the undeformed state ($0.44^\circ \,\pm \,4.48^\circ$) to the deformed state, for load case 1 ($0.49^\circ \,\pm \,2.24^\circ$) and load case 2 ($0.19^\circ \,\pm \,1.23^\circ$), we identified the underlying reason for the similar structural behaviour of the two models when external forces during passive stretching and initial fibre pre-stretch during isometric contraction are low. This extends to the dissimilar behaviour of the two models at higher values of external forces during passive stretching and higher initial fibre pre-stretch during isometric contraction, which is attributed to the increase in standard deviation of relative fibre inclinations during deformation of the muscle in these two load cases. We further investigated the influence of fibre inclination on the structural response of thermal simulation-informed models by computing additional designs from the metamodel with varying degrees of averaged angular deviations, i.e., $\bar{y}_{\bar{\Omega }}$. The selected design points exhibited an inverse correlation between relative fibre inclinations and $\bar{y}_{\bar{\Omega }}$, with the flattest fibre model resulting in $200\%$ higher displacement of the tendon end and $97\%$ lower reaction forces than the steepest fibre model during load case 1 and load case 2, respectively.

The study has several limitations. First, only the anterior aponeurosis of the *m. rectus femoris* muscle was considered. The posterior aponeurosis was not included since the primary focus of this work was identifying the aponeurosis that characterises the typical bi-pennate shape of the muscle. For the *m. rectus femoris*, this is indeed the anterior aponeurosis (Yang et al. 2006). However, the methods proposed in this work can be extended to predict the shape and position of the posterior aponeurosis by considering the entire *m. rectus femoris* as a RoI. Based on our results for the anterior aponeurosis, our procedure to identify the aponeurosis automatically can serve as a valuable image processing tool to segment inner structures of other muscles.

Second, the outer and distal regions of the muscle exhibit high angular deviations, i.e., $y_{\bar{\Omega }} \ge 45^\circ$ for the thermal simulation-informed model. We attribute these effects to (a) the absence of the posterior aponeurosis and its effects on the fibre orientation in the distal region and (b) differences in image resolution between MRI and DTI datasets, leading to an overestimation of the muscle RoI for determining fibre tractography from DTI. Nonetheless, our models demonstrate that despite such high local variations, the mechanical response of the DTI-based and thermal simulation-informed models is relatively similar, especially at low levels of passive and active deformation. In the future, the model should be further extended to incorporate the posterior aponeurosis to explore how it may influence fibre orientations and the mechanical response of thermal simulation-based fibre models.

Third, the mechanical response of the *m. rectus femoris* can be influenced by other anatomical structures in the thigh region that are not included in our model. These include interactions with other muscles within the same group, i.e., *quadriceps femoris*, or with other muscles, connective tissues, and subcutaneous tissues. Previous works by Röhrle and Pullan (2007) and Avci and Röhrle (2024) have shown that contact definitions between arm muscles vary and are crucial for accurate soft tissue deformation during passive stretch and isometric and concentric activation of skeletal muscles. Therefore, the model should be extended to include the effects of other MTA-complexes in the musculoskeletal system of the lower extremity.

Finally, the constitutive laws employed in this study are state of the art. The passive parameters of the material model are derived by fitting simulated values to experimental data on the macroscale, while muscle activity is represented by a homogeneous activation function. Micromechanical models exist, which derive the passive response of skeletal muscles through descriptions of mechanical structures on the micro-scale (Bleiler et al. 2019). Additionally, biophysical models (Heidlauf et al. 2013) can accurately describe the initial fibre pre-stretch in skeletal muscles, or chemo-electromechanical models (Heidlauf and Röhrle 2014) can represent complex and heterogeneous skeletal muscle recruitment functions. However, since this work focuses on identifying inner structures and fibre orientations of bi-pennate muscles and comparing the Laplacian-based approach for determining fibre angles with DTI-based fibre tractography, no further investigations into extending the constitutive model were made in this work.

The validation of this work would necessitate imaging data of the *m. rectus femoris* and its fibre orientation during active contraction and passive extension of the muscle. Dynamic ultrasound measurements can be employed to reconstruct the volume and fibre orientations of the muscle during skeletal muscle deformation (Sahrmann et al. 2024). Similarly, dynamic MRI sequences could provide insights into individual muscle length changes associated with the deformation of the *m. rectus femoris* during flexion and extension of the knee joint (Fiorentino et al. 2012; Qi et al. 2019). These measurements could be utilised not only to validate the FE simulations but also to generate additional optimisation criteria, such as numerical constraints and multi-objective goals, among others, to enhance the metamodel-based optimisation of thermal simulation-informed fibre models.

## Conclusion

In conclusion, we presented a novel method to determine the aponeurosis in bi-pennate muscles by determining the discrete *divergence* and the density map of the vector field of fibre tractography obtained from DTI sequences. The *m. rectus femoris* was used as an example. We utilised MRI and DTI datasets from a single subject to develop our approach and verified the results by demonstrating a strong agreement between the aponeurosis derived from our method and a manually segmented one.

In this work, we also introduced a metamodel-based approach using Laplacian-based, 3D thermal simulations to determine the fibre architecture in bi-pennate muscles. We showed that by optimising the boundary conditions (BCs) in a thermal simulation, angular deviations between DTI-based and thermal simulation-based fibre orientations can be minimised. We compared the two models, namely the DTI-informed and thermal simulation-informed fibre models during passive stretching and isometric activation, and concluded that (a) the thermal simulation-informed fibre models result in a homogeneous stretch field during deformation, (b) the structural response of the two models is similar, especially at low values of external load and initial fibre pre-stretch, (c) any difference in behaviour of the fibre models can be attributed to the relative fibre inclination, and (d) displacement of the distal tendon during passive stretching increases, while reaction forces at the proximal tendon during isometric contractions decrease with an increase in fibre inclination.

These findings suggest that both methods can be used to approximate the anatomy and physiology of bi-pennate muscles with reasonable accuracy. However, the extension of the automatic approach to identify the aponeurosis to other bi-pennate and multi-pennate muscles, as well as the application of metamodelling techniques to obtain fibre orientations for new subjects automatically, needs further investigation. Thus, future work should focus on conducting the study on a cohort of subjects to extend the metamodel with additional anatomical parameters and validate it as a predictive tool for determining fibre architectures in skeletal muscles without the need for acquiring DTI datasets.

## Data Availability

No datasets were generated or analysed during the current study.

## Footnotes

1 ANSYS LS-DYNA is an implicit and explicit FEM software and one of the most advanced simulation tools for nonlinear structural analysis.
